# Proactive and retroactive effects of novelty and rest on memory

**DOI:** 10.1177/17470218251346156

**Published:** 2025-05-21

**Authors:** Sumaiyah Raza, Judith Schomaker, Jörn Alexander Quent, Michael C Anderson, Richard N Henson

**Affiliations:** 1 MRC Cognition & Brain Science Unit, University of Cambridge, Cambridge, UK; 2 Department Health, Medical and Neuropsychology, Leiden University, Leiden, The Netherlands; 3 Department for Social and Behavioural Sciences, Leiden University, Leiden, The Netherlands; 4 Institute of Science and Technology for Brain-Inspired Intelligence, Fudan University, Shanghai, China; 5 Key Laboratory of Computational Neuroscience and Brain-Inspired Intelligence (Fudan University), Ministry of Education, Shanghai, China; 6 MOE Frontiers Center for Brain Science, Fudan University, Shanghai, China; 7 Department of Psychiatry, University of Cambridge, Cambridge, UK

**Keywords:** Novelty, Wakeful Rest, Consolidation, Behavioural Tagging, Memory, Temporal Distinctiveness

## Abstract

Novel experiences appear to benefit memory for unrelated information encoded shortly before or after. Other research suggests that memory is impaired by effortful tasks following encoding, compared to simply resting. This registered report explicitly tested the proactive and retroactive effects of novel exploration and wakeful rest. Four groups of participants explored a novel or familiarised virtual environment, either shortly before or shortly after encoding a list of unrelated words. A fifth ‘wakeful rest’ group performed a low-effort attention task before and after encoding. Memory was tested with immediate free recall, delayed (next day) free recall and delayed recognition with confidence judgements (from which recollection and familiarity were estimated). Bayes factors provided evidence against both proactive and retroactive benefits of novelty across all measures of memory, but provided evidence for a retroactive benefit of rest for immediate recall. In exploratory analysis, we also found evidence for a proactive benefit of rest on immediate recall. We argue that the bidirectional benefits of wakeful rest are more easily explained by Temporal Distinctiveness theory than Consolidation theory. Overall, wakeful rest surrounding learning may represent a useful intervention for improving memory, while novel exploration may not.

## Introduction

Research with rodents has suggested that novel experiences can facilitate long-term memory for unrelated stimuli encoded close in time to the novel experience. Typically, rats are exposed to a weak version of a training protocol, such as contextual fear conditioning, inhibitory avoidance training or spatial object recognition (e.g. Ballarini et al., 2009; Justel et al., 2021; Moncada & Viola, 2007). If the rats also explore a novel environment shortly before or after the training, they show improved memory for this training when tested 24 h later, compared with rats that did not explore a novel environment. This novelty-related memory enhancement has also been called ‘behavioural tagging’ (Moncada & Viola, 2007; Moncada et al., 2015). According to Behavioural Tagging Theory (BTT; Moncada & Viola, 2007; Moncada et al., 2015; see Dunsmoor et al., 2022 for an in-depth review), novelty induces plasticity-related neurochemical changes that facilitate consolidation of unrelated encoding that happens to occur in close temporal proximity to the novel experience.

Novel experiences also seem to have a comparable effect in humans. For example, Schomaker and colleagues developed a paradigm in which participants explore a novel virtual reality (VR) environment shortly before encoding a list of words. They found that recall of these words after a short delay was better when participants had explored a novel environment compared to an environment with which they had previously been familiarised (Schomaker & Wittmann, 2021; Schomaker et al., 2014, 2022). The memory enhancement was not accounted for by differences in subjective arousal following exploration. In another, more ecological example (Ballarini et al., 2013), school children were tested on their memory for a story heard the previous day. Children who had received a novel lesson 1 h before or 1 h after hearing the story were found to have better memory for it than children who did not receive a novel lesson. The memory benefit was not seen when the children had been familiarised with the lesson twice before. Nor was the benefit seen in children who received the novel lesson 4 h before or after hearing the story, consistent with the plasticity-related changes induced by the novel experience, only lasting for a limited time window. The novel lessons were conceptually unrelated to the story content, and the effect was comparable whether the novel lesson was about science or music, suggesting that the content of novel experiences is not important. With a similar paradigm, Ramirez Butavand et al. (2020) showed that the memory improvement associated with novel lessons persisted for up to 45 days, supporting the robustness of the effect and the potential utility of novel experiences as a tool for improving memory. Taken together, these results support the existence of novelty-related memory enhancement in humans, in keeping with rodent research (though other factors, like the semantic relationships between stimuli, may be especially important in humans – Dunsmoor et al., 2022).

However, using a paradigm similar to that of Schomaker and colleagues, a recent registered report by Quent and Henson (2022) failed to replicate the effect of a novel VR experience on memory for words. During an initial, incidental study phase, participants made semantic (deep) or orthographic (shallow) judgements about words. This was followed by a novel experience with immersive VR (iVR), in which participants used a headset to look around a virtual kitchen to find 20 objects, and their memory for the location of these objects was subsequently tested (none of the participants had used iVR before). Immediately after the iVR, participants freely recalled the words studied before the iVR experience, and then the next day, completed a recognition test for the words, combined with a ‘remember/know’ judgement to separate recollection and familiarity-based memory (Tulving, 1985). A separate group of participants completed the same procedure, except they had performed the identical iVR task on the preceding day, so that it was less novel. Bayes factors showed evidence for no difference between groups in memory for the words, in either the immediate recall or delayed recognition test (collapsed across deep/shallow study task and across recollection or familiarity, with no evidence for or against any interactions with these factors). This suggests that, at least in this version of the paradigm, the novel iVR experience did not retroactively enhance memory, challenging previous research.

However, there are some key factors that may explain the divergent results of Quent and Henson (2022) and Schomaker et al. (2014). For example, a later study by Schomaker et al. (2021) suggested that active navigation within virtual environments (VEs) is important for the novelty effect (consistent with the original animal studies, which allowed rodents to explore the novel environment). Furthermore, Schomaker et al. (2022) found that exploration behaviour within their relatively large VEs, indexed by roaming entropy, predicted recall performance. By contrast, the single, small (~5 × 4 virtual metres) room used by Quent and Henson (2022) required negligible navigation/exploration. However, this finding may be specific to novel ‘spatial’ experiences, as a number of other studies have found a benefit of novelty with no requirement for exploration (e.g. Abrahan et al., 2020; Ballarini et al., 2013; Bunzeck & Düzel, 2006; Fenker et al., 2008). Nevertheless, in the experiment reported here, we used a ‘spatial’ novel experience, and so we employed the relatively large VEs used by Schomaker et al. (2022).

A second factor offered by Quent and Henson (2022) to explain the divergent results – and the one most relevant to the current proposal – is that, whereas the studies by Schomaker et al. tested (and found) a proactive effect of novelty, Quent and Henson tested (and failed to find) a retroactive effect. Similarly, two other studies failed to find a retroactive effect of novelty in adults (McClay & Dunsmoor, 2018) and typically developing children (Baumann et al., 2020; though note they did find a retroactive novelty effect in those children with attention deficit hyperactivity disorder). Despite the previous findings in rodents and school children of both pro and retroactive effects, it is possible that novelty only exerts a proactive effect in this type of VR paradigm. Indeed, one reason for the lack of a retroactive effect of novelty may be an additional, counteracting, detrimental effect of performing a cognitively demanding task shortly after encoding the words. This is because another literature has suggested that cognitively demanding tasks impair consolidation of previously encoded memories, relative to wakeful rest, as briefly reviewed next.

Several previous studies have shown that effortful tasks following encoding can impair memory. For example, Dewar et al. (2012) found that older adults had worse story recall when they engaged in an effortful visual discrimination task for 10 min following encoding, compared with when they rested for 10 min. This performance difference was evident after delays of 15 min, 30 min and 7 days. Similar beneficial effects following 9 to 10 min of post-encoding rest have also been reported in young adults (Craig et al., 2014; King & Nicosia, 2022), patients with amnesia (Cowan et al., 2005; Dewar et al., 2009) and patients with amnestic mild cognitive impairment (Alber et al., 2014). It seems unlikely that this benefit was due to intentional rehearsal of encoded information during the resting period, as the effect also occurred for recognition of ‘unrecallable’ words (i.e. foreign names; Dewar et al., 2014). Furthermore, the fact that the effortful tasks used in these studies were non-verbal and unrelated to the encoded information suggests that impaired memory reflects the cognitive load of the task, rather than interference from competing stimuli (e.g. at retrieval). A meta-analysis of 10 similar studies found a significant, moderately sized benefit of post-encoding rest on verbal memory (Cohen’s *d* = 0.38) (Humiston et al., 2019). These retroactive effects of cognitive effort are often explained in terms of Consolidation theory (see Wamsley, 2022): The theory that memory traces gradually become transformed after encoding, through cellular structural changes and system reorganisation, and until they are consolidated in this way, they remain susceptible to disruption (Müller & Pilzecker, 1900; Squire et al., 2015). Assuming these consolidation processes require cognitive/neural resources, then they will be impaired by effortful tasks (Wixted, 2004). As the novel VR task used by Quent and Henson (2022) was quite effortful (involving intentional learning of the location of objects in the room), it may have impaired consolidation of memories for the preceding words, counteracting any benefit of the novelty of the iVR task. Even if active navigation around Schomaker et al.’s larger VE is also effortful, because consolidation processes are ‘asymmetrical’ (can only occur after encoding), no such masking of the novelty effect would occur in Schomaker et al.’s proactive paradigm.

**Figure 1.**
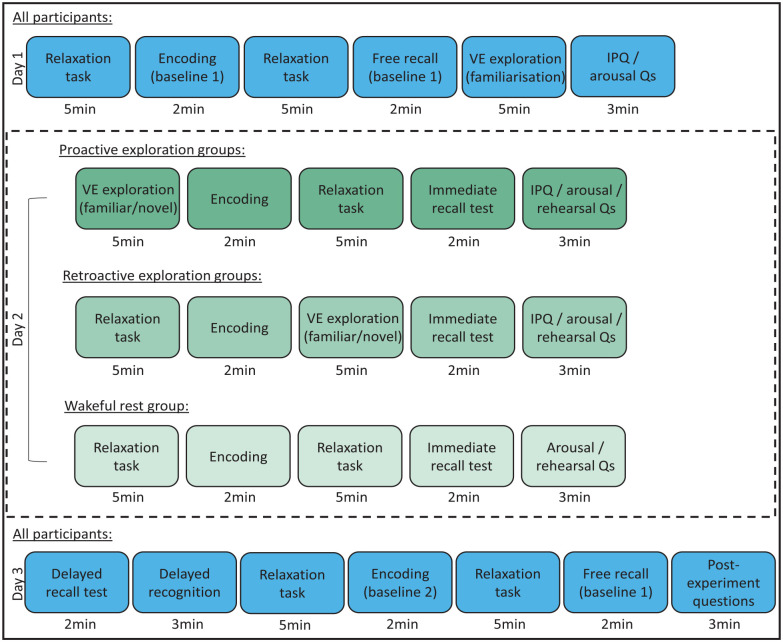
Order of experimental tasks for each group. Day 1 consisted, for all participants, of the first baseline encoding and free recall phase, followed by familiarisation with a VE. Day 2 involved one of the interventions (exploration of novel VE, exploration of familiar VE or wakeful rest – ‘relaxation task’, depending upon the participant’s experimental group), encoding and free recall test phases. Encoding occurred prior to VE exploration for the retroactive groups and after VE exploration for the proactive groups. This session ended with a subjective rating of arousal, questions about how often participants thought about the words during the intervention, and the Igroup Presence Questionnaire (IPQ) for the exploration groups only. Day 3 began with a delayed free recall test for the words learned on the previous day, followed by a recognition test for these words, a second baseline encoding and free recall phase, and finally some questions regarding participants’ lifestyle and subjective experience of the experiment. Delay periods throughout were filled with the relaxation task to match the time between encoding and test across proactive/retroactive groups, and the task structure across days. There were approximately 5 min of written task instructions at the start of each session, and an opportunity to report technical issues at the end of each session. *Note*. VE = virtual environment.

To test this possibility, we compared the effects on memory of (a) exploring a novel VE, (b) exploring a VE that has been familiarised the previous day and (c) spending the same amount of time in ‘wakeful rest’, that is, performing a very undemanding, unrelated task. Furthermore, we explicitly tested both proactive and retroactive effects of novelty: Some participants explored the VE before studying a list of words (‘proactive’ groups, as in Schomaker et al.’s studies), whereas others explored the VE after studying a list of words (‘retroactive’ groups, as in Quent & Henson’s study). To match the retention interval before testing memory, the proactive groups performed the wakeful rest task after study, while the retroactive groups performed the wakeful rest task before study (see ahead to Figure 1). Thus, there were five groups in total: proactive novelty group, retroactive novelty group, proactive familiarised group, retroactive familiarised group and the ‘wakeful rest’ group who performed the undemanding task before and after studying words.

Memory was tested with immediate free recall, 24-h delayed free recall and 24-h delayed recognition with confidence ratings. The inclusion of both immediate and delayed memory tests allowed us to address a key difference between rodent and human research on novelty effects. Rodent studies typically test memory after an extended delay (e.g. 24 h), based on the BTT assumption that protein synthesis-dependent consolidation processes that are facilitated by novelty, take place over several hours after encoding (Moncada et al., 2015). However, many human studies (e.g. those by Schomaker et al.) test memory after only short delays (e.g. 10 min). If novel exploration or wakeful rest affect consolidation processes, it is possible that their effects on memory would be greater on delayed tests. However, in case free recall performance after 24 h was too close to the floor, we also tested delayed recognition. Furthermore, we used confidence ratings on the recognition memory task to estimate recollection versus familiarity processes, by applying the independent, dual-process model of Yonelinas (1994) to the resulting Receiver Operating Characteristic (ROC) curves. This distinction between recollection versus familiarity was important because BTT claims that the novelty-induced neurochemical changes must occur in the same neural population that is encoding the stimuli (Moncada & Viola, 2007; Moncada et al., 2015). Given that exploration of novel environments is likely to engage the hippocampus, and that the hippocampus is associated with recollection but not familiarity (Aggleton & Brown, 1999; Argyropoulos et al., 2022), the effects of novelty were expected to be found on estimates of recollection but not of familiarity. This is consistent with previous studies (e.g. Schomaker et al., 2014) that have found a novelty effect on recall but not recognition, assuming that their recognition data were dominated by familiarity.

In summary, we employed a between-participant, two-way factorial design that crossed novel versus familiar exploration with pro versus retroactive order, plus an additional control group experiencing wakeful rest only. Our dependent variables were (a) immediate free recall, (b) delayed free recall and (c) delayed recognition memory split by recollection versus familiarity. Our primary aims were to (a) replicate Schomaker et al.’s (2014) findings that exploration of a novel versus familiar VE benefits immediate recall of unrelated words and (b) replicate Dewar et al.’s (2012) findings that wakeful rest retroactively benefits immediate and delayed memory compared to a more effortful task (exploring a familiar VE).

Regarding our first aim, BTT predicts a novelty-related memory benefit in both proactive and retroactive groups, and this benefit should be greater on memory tests that are delayed and that involve recall or recollection. Regarding our second aim, Consolidation theory predicts that wakeful rest benefits memory retroactively, but not proactively, and this effect should again be greater for delayed free recall or recollection during a delayed recognition test, assuming only hippocampally dependent memory (rather than familiarity-based memory) undergoes consolidation (McClelland et al., 1995). Importantly, if both theories are true, the retroactive effect of novelty might be small or absent, owing to the difference between novel and familiar VEs being masked by the detrimental effect of performing any effortful task after encoding (and potentially explaining the lack of a retroactive novelty effect in the study by Quent & Henson, 2022).

Finally, after the immediate recall test, for the groups experiencing the exploration interventions, we assessed participants’ subjective feelings of immersion/presence within the VEs. This was to address another apparent discrepancy in the literature: Schomaker et al. (2014) found that these ratings positively predicted recall performance in their proactive paradigm, whereas Baumann et al. (2020) found that these ratings negatively predicted recall in their retroactive paradigm. One possibility is that, in the proactive paradigm, greater immersion in the VE led to greater improvements in arousal and motivation during the subsequent encoding and test phases, in turn, leading to better memory. Conversely, in the retroactive paradigm, greater immersion (which has been suggested to involve greater recruitment of cognitive resources; Barreda-Ángeles et al., 2021) may have caused greater impairment of consolidation after encoding and thus poorer memory performance, which would be in line with our theorised interaction between novelty and cognitive effort. We also measured reaction times (RTs) during the encoding tasks (pleasantness judgements), in case these were speeded by increased arousal following novel exploration (in the proactive group).

In summary, the results of this study test the claims of neurocognitive theories like BTT and Consolidation theory, thereby improving our understanding of the mechanisms underlying long-term memory, as well as evaluating two potential interventions for improving memory; important for potential clinical and educational applications.

## Procedure

Informed consent was obtained, and participants were tested online, once on each of 3 successive days. Participants were randomly assigned to one of the five experimental groups. The order and duration of experimental tasks for each group are depicted in Figure 1.

Participants could only complete the experiment using a Windows PC or laptop (i.e. not using a mobile device or other operating system), on a Chrome browser and with an internet speed surpassing 5 mbps – these criteria were assessed programmatically and were included to ensure technical compatibility with the web experiment and the VE software. Detailed instructions for each task were given at the start of each session. Participants were instructed to complete all sessions of the experiment in the same quiet setting, at the same time each day. At the start of the experiment, they reported the type of setting they were in by selecting from some options (i.e. familiar, quiet/familiar, distractions present/novel). The experiment only continued if they were in a familiar setting, free from distraction, to reduce extraneous novelty effects. The initial task instructions included a comprehension test. If participants failed the comprehension test twice, they were required to contact the experimenter to help them understand the instructions via email or direct message before taking part. Only two participants who were included in the final sample did this.

Participants experienced three encoding phases throughout the experiment – one per session. The procedure for each was identical. During these, participants viewed words sequentially and decided for each whether they found it pleasant or unpleasant. They were instructed that there were no correct or incorrect answers. Words were presented in a random order, for 3,000 ms each (300 ms of intertrial interval), and pleasant/unpleasant responses were given using the ‘F’/‘J’ keys. Throughout the task, words were presented inside a coloured box – the colour differed for each of the three encoding tasks participants undertook. Participants were informed that their memory for words would be tested, but that they should focus on the task shown on-screen, to minimise the likelihood that they would cheat by writing down words or employ their own strategies for memorising words. There were four practice trials as part of the instructions on Day 1. One word list was used for each encoding phase, and the assignment of lists was counterbalanced across participants for each encoding phase.

There were four free recall tests during the experiment. The procedure for each was identical, and the test always targeted the word list from the most recent encoding task: encountered earlier in the same session for the Immediate Recall, Baseline 1 and Baseline 2 tests; and encountered 24 h prior for the Delayed Recall test. Participants had to type in as many words from the previously learned list as possible, within 2 min. The same coloured box from the encoding phase of the relevant list framed the response box, to help participants differentiate the encoding phases. Recall tests always targeted the most recently learned list only; participants were never asked to recall words from multiple lists. The recall data were scored by counting the number of correctly recalled words from the list associated with each test and dividing this number by the total words (16) to give the proportion correctly recalled. Words reported with typing errors (up to two letters incorrect or mixed up) were coded as correct unless the error resulted in a different meaningful word (e.g. ‘well’ given in place of ‘wall’).

Across the three sessions, this resulted in three study-test blocks: a pre-encoding task, encoding, a post-encoding task, followed by free recall. The key between-participant manipulation involved the study-test block on Day 2. The pre- and post-encoding tasks were either an undemanding wakeful rest period, or the novel or familiar VE exploration task, depending on the experimental group. The 24-h delayed recall task and the recognition task (both on Day 3) also targeted the words learned as part of this block. The Baseline 1 (on Day 1) and Baseline 2 (on Day 3) study-test blocks both involved an undemanding wakeful rest period during the pre- and post-encoding phases. Note, this means that the procedure was identical for Baseline 1, Baseline 2 and Immediate Recall for the Wakeful Rest group.

The undemanding wakeful rest periods involved the ‘relaxation task’, which was adapted from the study by Dewar et al. (2010). Participants were instructed to relax, listen to the ‘waterfall’ sound, gaze at a central fixation cross on the screen, and press the space bar when they heard piano notes or saw the fixation cross temporarily change colour. This low-effort task was to reduce the extent to which participants rehearsed words or engaged in autobiographical thinking, which could introduce additional interference (Craig et al., 2014). It is possible that this task introduced small deleterious effects on memory compared to complete rest, but, given that participants were unsupervised, this task afforded greater control and a way of checking that participants were not engaged in other activities. Participants were also explicitly instructed on how important it was to focus on this task, rather than check their phones, for example, in order for their data to be informative (we believe the majority of participants want their data to be useful). A similar tone-detection task has been shown not to significantly disrupt delayed recall performance compared with complete wakeful rest in healthy adults (Dewar et al., 2010).

During VE exploration (VE familiarisation on Day 1 for all groups and the novel and familiar interventions on Day 2 for the four exploration groups), participants explored one VE for 5 min, moving forward using the W key and changing heading direction using the mouse or trackpad. They were instructed to try to stick to paths. Participants in all groups completed VE familiarisation on Day 1 (half of the participants were familiarised with one VE and half with the other VEs). For participants undergoing the novel and familiar exploration interventions, one of the relaxation tasks before or after encoding (proactive/retroactive groups) was replaced with VE exploration. The familiar groups explored the familiarised VE for 5 min, and the novel groups explored the unseen VE.

Given the 6 possible orders in which the 3 word lists could be encoded, combined with the counterbalancing of VE familiarisation, each group required multiples of 12 participants.

At the end of the session on Day 2, participants answered some questions relating to the intervention they experienced (either VE exploration or the relaxation task). All rated their arousal following the intervention using a sliding scale from 1 to 9 (1 = *very calm*, 9 = *very excited*). Participants in the proactive intervention groups and in the wakeful rest group rated how often they thought about the words they learned during the intervention/relaxation task. Participants who experienced the exploration interventions also answered the 13-item IPQ (Schubert et al., 2001) to measure their subjective feelings of immersion in the VR experience.

During the recognition test, participants were shown the list of 16 words encoded on the previous day (Day 2), interspersed with 16 lures. Words were shown sequentially, in a random order, and remained on screen whilst participants indicated how confidently they did or did not recognise a word (i.e. confident new/unsure new/unsure old/confident old). For each participant, we totalled response frequencies at different confidence levels for lures and targets separately. We then used a MATLAB toolbox (Koen et al., 2017) to generate ROC curves and fit a well-established model of Yonelinas (1994) model to each curve, estimating two parameters for each participant: one reflecting the probability of recollection, and the second, a signal detection measure of continuous familiarity (*d*’).

At the end of each session participants completed an ‘instructional manipulation check’ (as described by Oppenheimer et al., 2009) in which they had to follow explicit instructions to respond to a simple question (e.g. ‘When asked to choose a colour, you must select green. This is an attention check. Based on the text above, which colour do you choose? [Select from options green/blue/red/yellow]’). At the end of the experiment, participants were also given a ‘seriousness check’ (Aust et al., 2013). As part of this, they were reminded of our need for good quality data, prompted to report whether they participated seriously and asked to provide any reasons their data should not be used. The instructional manipulation and seriousness checks were used to help maintain high standards of data quality.

To conclude the experiment, participants answered some questions about their gaming habits (e.g. ‘How often do you play online first-person view games?’). This data were to provide additional insight into potential mediators of the Novelty effect.

## Stimuli

**Figure 2.**
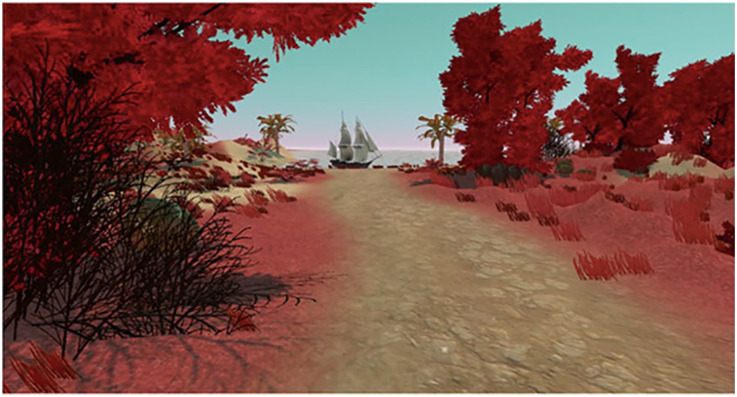
One of the VEs used in the exploration interventions (with thanks to Milan van der Kuil for VE design). *Note*. VE = virtual environment.

The web experiment was built using jsPsych version 7.3, a JavaScript toolbox for creating behavioural experiments (de Leeuw et al., 2023), and hosted on JATOS (Lange et al., 2015). The exploration interventions utilised two VEs from Schomaker et al. (2022) consisting of fantasy lands. These were created in Unity Version 2017.2.21f1 (unity.com) and matched for size, path length and number of intersections. Each environment contained marked paths through brightly coloured foliage and unusual landmarks (Figure 2). Participants downloaded the Unity files and ran them from their own laptop or PC. During exploration, the 3D coordinates of the moving agent within the VE were logged for all timepoints with a sampling rate of 15 Hz. The relaxation task used a 5-min recording of ‘brown noise’ (sounds like a waterfall) with 3 to 6 piano notes embedded and 3 to 6 screen colour changes occurring at random timepoints. For the word encoding tasks, 64 English concrete nouns taken from Otten et al. (2001) were divided into 3 lists of 16 words, plus 16 lures for use in the final recognition test. The lists were selected so as not to differ in terms of characteristics available in the MRC Psycholinguistic Database (Wilson, 1987).

## Statistical design and hypotheses

Inferences were based on Bayes factors (BFs) for the alternative (H1) versus null (H0) hypotheses. We regarded a conclusive outcome as a *BF* for H1 (‘*BF*_10_’) or *BF* for H0 (‘*BF*_01_’) exceeding 6, based on the journal criteria. All registered hypotheses were tested with one-tailed, unpaired *T*-tests unless otherwise specified. BFs were calculated for *T*-tests using the ‘ttestBF’ function from the ‘BayesFactor’ package in R with a ‘medium’ value for the ‘rscale’ hyperparameter (default √2/2). Bayesian linear models used in exploratory analyses were fitted using the ‘lmBF’ function from the same package, and Bayesian ordinal models were fitted using the ‘brm’ function from the ‘brms’ package.

Our two main Registered Hypotheses were as follows: (a) ‘Proactive Novelty effect’ – that recall would be better in the proactive novelty group versus the proactive familiarised group (i.e. replicate Schomaker et al., 2014; and support BTT) and (b) ‘Retroactive Resting effect’ – that recall would be better in the wakeful rest group versus the retroactive familiarised group (i.e. replicate Dewar et al., 2012; and support Consolidation theory). These main hypotheses were evaluated with immediate free recall performance (i.e. group mean number of correct recalls on the Day 2 test). Our stopping rule to terminate our sequential design (see ‘Participants’ section below) was based on obtaining conclusive evidence for both of these hypotheses with immediate recall data (i.e. BF > 6 for either the null or alternative), or if we recruited the maximum realistic sample size (see ‘Participants’ section) before obtaining conclusive evidence.

After terminating data collection, we conducted secondary analyses examining the ‘Retroactive Novelty effect’ (Registered Hypothesis 3) by comparing immediate recall in the retroactive novelty group with the retroactive familiarised group. If a bidirectional Novelty effect existed, then recall would be better in the novelty group; however, if a Retroactive Resting effect also existed, then there may have been no difference in recall between these groups. This latter pattern would support our theorised interaction between novelty and cognitive effort and explain the divergent results of Schomaker et al. (2014) and Quent & Henson (2022).

For analyses involving immediate free recall, we also tried subtracting (registered) and regressing out (not registered; see ‘Deviations from stage 1 registration’ section) participants’ baseline scores to adjust for individual differences in memory ability.

We also registered analyses assessing the Proactive Novelty effect, Retroactive Resting effect and Retroactive Novelty effect for other measures of memory. The same pattern of results was expected for delayed free recall performance and for recollection on the delayed recognition test. However, for familiarity on the recognition test, we expected no effect of Novelty or Resting as explained above (see ‘Introduction’ section).

Finally, we registered follow-up analyses that were dependent on the results of our main hypothesis tests. If we found a Novelty effect (proactive and/or retroactive), regression analyses would be conducted across participants to relate the size of this effect to measures of (a) exploration (i.e. ‘roaming entropy’ – Schomaker et al., 2022), (b) subjective ratings of arousal and (c) IPQ scores. Furthermore, if we only found evidence of a Proactive Novelty effect, a one-tailed, unpaired *T*-test would be conducted to check whether response times on the encoding task differed following the novel versus familiar intervention; significantly faster responses following the novel intervention would suggest that increased arousal/motivation could be responsible for the Proactive Novelty effect.

## Participants

We employed a Bayesian Sequential Design whereby we continually recruited participants until we obtained conclusive evidence for H1 or H0 (i.e. *BF*_10_ or *BF*_01_ > 6) for both of our two main hypotheses, or until we reached a predetermined maximum sample size based on resource constraints. In our case, our maximum sample size was 168 per group (i.e. *N* = 840 participants total). To allow full counterbalancing (see ‘Procedure’ section), we recruited participants in batches of 12 per experimental group.

Prior to commencing recruitment, we determined the ‘power’ of our design by performing 10,000 simulations of the two one-tailed, unpaired Bayesian *T*-tests that our stopping rule was based on. We used effect sizes (a) taken from previous literature for H1 or (b) set to zero for H0. For Hypothesis 1 (Proactive Novelty effect), we set Cohen’s *d* = 0.44, based on half that of Schomaker et al. (2014). We halved the reported effect size to account for potential effect size inflation, for example, due to publication bias, because the estimate is based on only one study. For Hypothesis 2 (Retroactive Resting effect), we set Cohen’s *d* = 0.38, based on a meta-analysis of 10 studies investigating post-encoding rest (Humiston et al., 2019). These simulations informed us that, with a maximum *N* of 168 per experimental group, our design was well powered (i.e. ~80% power) to detect both of our main effects of interest if they both existed (Novelty effect/Resting effect) and had a very low chance of producing misleading evidence (i.e. <4% false positive rate). See Supplemental Material for this power analysis.

This study is of the type approved by the Cambridge Psychology Research Ethics Committee (PRE.2020.018). Participants were recruited using Prolific (www.prolific.co) and the MRC Cognition and Brain Sciences’ SONA system, in-house participant panel. They were paid at the end of the 3 days at a standard rate of £6/h with each session rounded up to the nearest 15 min (i.e. £3 per day = £10 total), plus a bonus of £5 after Day 3 to encourage completion of all sessions. All participants self-reported that they were 18 to 40 years old, had normal or corrected-to-normal vision, had normal hearing, were fluent in English and had no history of diagnosed neurological or psychiatric illness.

## Data quality checks and exclusions

### Online checks

There were some pre-specified circumstances under which a participant’s involvement in the study was terminated prior to completion. These were monitored automatically by the web experiment program. Ideally, participants would have been tested over 3 consecutive days with 24 h between each session, however, participants with a delay of up to 48 h between the first and second sessions were still included in the study. Those with a delay of longer than 30 h between the second and third sessions, or who did not complete the study within 4 days had their involvement terminated. Participants were not able to complete the next session before at least 20 h had passed. Furthermore, participants’ involvement was terminated if they failed two instructional manipulation checks throughout the study. Termination also occurred if: Accuracy was less than 70% on a relaxation task, if participants made 70% more responses than required on a relaxation task (to prevent participants continuously responding throughout the task), or if participants responded in less than 150 ms to more than 70% of trials on an encoding task. Out of 2,268 participants who signed up for the study and completed the first task, 224 had their involvement terminated for one of these reasons. Sixty-eight (out of the 224) were terminated in the final session, and so they were included for further data quality checks because their data were still usable if they had completed the delayed recall task (i.e. they may have only been missing Baseline 2 recall data). Overall, we saw a termination rate of 9.88% out of all study attempts. Eight hundred and eighty-seven participants completed the essential parts of the experiment (i.e. provided both immediate and delayed recall data), leading to a combined termination and dropout rate of 39.11%.

### Offline checks

Data for the 887 participants reaching the final session were taken forth for preregistered offline quality checks. These checks were carried out after each new batch of participants had been recruited. At this stage, 69 participants were excluded because they failed the following criteria: attempting the experiment more than once (*n* = 4), self-reporting that they did not take the experiment seriously (*n* = 3), providing reasons at the end of each session which indicated their data would not be usable (*n* = 3, all reporting that they ran the explore task more than once), spending longer than 5 min on the instruction page of a relaxation task where this would have disrupted the critical intervention–encoding–intervention–recall task flow (*n* = 1), navigating away from the experiment tab during the relaxation task (*n* = 0), missing explore task result files (required to be uploaded manually by the participant, *n* = 19), having explore task data covering less than 270 s (only relevant for first two batches as the explore task ‘quit’ button was disabled after this point so that the task always ran for 5 min, *n* = 5), spending more than 5 min on the encoding task instruction page (*n* = 11), repeating the Day 2 encoding task due to a bug in the web experiment code (*n* = 12), spending more than 5 min on the recall task instructions page (*n* = 11). Overall, we saw a 7.78% exclusion rate. Note, these reasons were assessed in a sequential manner in the order listed, meaning that any participant excluded because, say, they reported not taking the experiment seriously, would not be assessed for having missing exploration files. Where these exclusion criteria affected only the Baseline 1 or 2 test, only the affected baseline test data were excluded (i.e. ‘partial exclusions’ otherwise included in final sample; *n* = 19). Altogether, this produced 818 usable datasets. The final sample had to be a multiple of 12 due to counterbalancing groups, so 744 datasets were selected across all groups for inclusion in the final sample based on the earliest recruited correct counterbalancing permutation.

### Outlier removal

To identify outliers, we pooled the task data across groups and computed the inter-quartile range (IQR) for each relevant score. For VE exploration we used the total distance travelled by each participant in each exploration task (summed Euclidean distance between coordinates visited). We set the cut-off to be 1.5 × IQR below the lower quartile (484.97 arbitrary units) to remove participants who explored the least. Twenty-eight participants were removed this way. For the recall data, we used each participant’s mean immediate recall score (i.e. the mean of: Baseline 1, Immediate recall and Baseline 2 scores) and set the cut-offs to be both 1.5 × IQR below the lower quartile or above the upper quartile to remove any participants who did not remember enough words or those who may have ‘cheated’ (cut-offs = 0.05–0.67 proportion of words recalled). Twelve participants were removed this way. New participants were recruited to replace these 40 outliers, and the same cut-offs were applied to ensure adequate performance. Thus, 4.89% of the 818 usable datasets collected were deemed outliers.

## Deviations from stage 1 registration

1. We originally said, ‘To discourage participants from multitasking, they will be prompted to complete the experiment in full-screen mode, and an alert box will appear if they exit this mode’. However, we were not able to execute this in the web experiment due to technical constraints. Nonetheless, we felt that the existing online data quality checks would ensure participants were adequately engaged throughout.
2. The following inclusion criteria were not registered but were necessary to ensure technical compatibility with the web experiment and VE software: Windows PC or laptop (i.e. not a mobile device or other operating system), Chrome browser and internet speed exceeding 5 mbps.
3. We originally said, individuals would be ‘excluded and replaced with a new participant if they spend less than 0.5 s on instruction pages’. We did not do this because the instructions were broken down across many pages, so there was very little text on each page. They were also designed so that participants would become very familiar with the instructions for each task separately. Therefore, it was not necessarily problematic for participants to spend very little time on any single instruction page. We felt the inclusion of a comprehension test on the first day sufficiently ensured participants had paid attention to the instructions and had an adequate understanding of the tasks.
4. After commencing data collection, we received some feedback that non-native English speakers may not be familiar with 10 of the words. These words were replaced so that the lists were unchanged in terms of psycholinguistic characteristics. See Supplemental Material for the word lists. Only 32 participants completed the experiment with the original set of words, who were approximately equally distributed across the groups and were all native English speakers; we received no comments from the remaining 712 participants to say that they were unfamiliar with any of the words in the final stimulus set. The order of the lists was counterbalanced so that the findings of the study were not specific to a particular set of words.
5. We said we would apply a 1.5 × IQR threshold to the relaxation task performance data as part of our outlier removal process, but we chose not to do this because we had already applied a 70% performance threshold on this task as part of our online data quality checks.
6. We said we would determine outliers based on 1.5 × IQR for ‘overall recall performance pooled across the free recall tests on all days’ – implying the inclusion of both Immediate and Delayed recall scores. However, we chose not to include Delayed recall scores because the Delayed recall scores skewed participants’ mean overall recall score to a greater extent when they did not have both baseline scores (i.e. partial exclusions, *n* = 19). See Supplemental Material for an alternative approach to removing outliers that included Delayed recall outliers – results did not differ from the main paper.
7. We preregistered an exploratory analysis where we would ‘try subtracting each participant’s baseline recall performance, averaged across the first and last days’. However, the within-participant correlation between the two baseline scores was low (*r* < .5) and we were concerned that we would introduce additional noise through this subtraction. Instead, we regressed out the effect of baseline performance for better sensitivity (Van Breukelen, 2006), and we did this using only Baseline 1 scores because there were no procedural differences between groups at this point in the experiment.

## Results

In accordance with the stopping rule for our sequential design, we terminated recruitment for the proactive novelty and proactive familiarised groups after 10 batches of participants (*n* = 120 per group) because we obtained conclusive evidence for our first hypothesis at this point. For the remaining groups, we terminated recruitment after 14 batches of participants (*n* = 168 per group), because we obtained conclusive evidence for our second hypothesis (this number also happened to be the pre-specified maximum realistic sample size). This means that not all experimental groups had the same number of participants; however, none of our confirmatory contrasts were between groups of a different size (note that this decision was agreed upon by the Editor). As a result, the final analysis included *N* = 744 participants (fully counterbalanced within each group).

### Confirmatory hypotheses

#### Immediate and delayed recall

**Figure 3.**
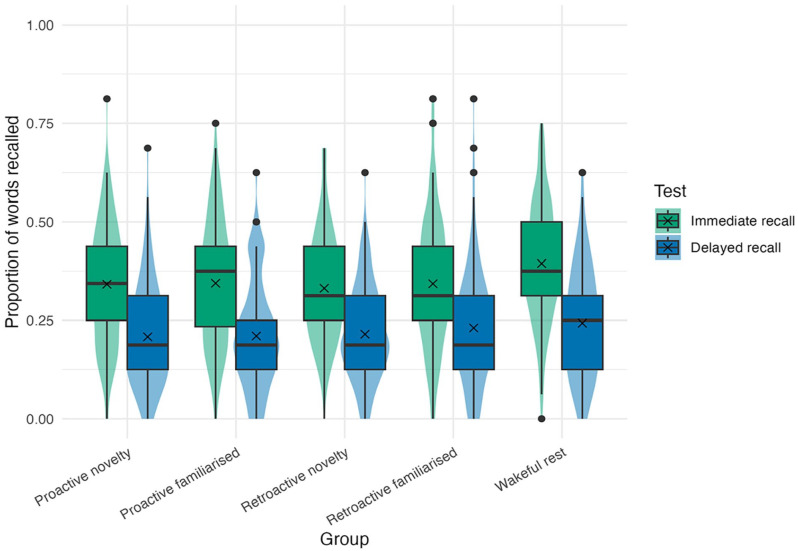
Violin plots and boxplots for immediate and delayed recall data across groups. Note that our inferential statistics are based on Bayesian *T*-tests, which assess differences between group means (represented by *X*s). Also note that each group contains different numbers of participants (*n* = 120–168); see beginning of ‘Results’ section. Dots represent recall scores falling outside 1.5 × IQR of each group and test type separately. These data points were included in the main analysis because they were not outliers when pooling immediate recall scores across all conditions, which was our registered approach to avoid biasing differences between groups (see ‘Outlier removal’ section above, and Supplemental Material for analysis with outliers removed by group).

The results of each type of recall test are summarised in Table 1, and the distributions of immediate and delayed recall data across groups are displayed in Figure 3. BFs for our two main hypotheses for the Proactive Novelty Effect (H1) and the Retroactive Resting Effect (H2), as well as our secondary hypothesis about the Retroactive Novelty Effect (H3), are shown in Table 2.

**Table 1.** Mean (±standard deviation) proportion of words recalled in each type of test, by group.

Experimental group	Immediate recall	Delayed recall	Baseline 1	Baseline 2
Proactive Novel	0.34 (±0.15)	0.21 (±0.13)	0.38 (±0.15)	0.33 (±0.15)
Proactive Familiar	0.34 (±0.15)	0.21 (±0.13)	0.40 (±0.15)	0.36 (±0.16)
Retroactive Novel	0.33 (±0.14)	0.22 (±0.12)	0.41 (±0.14)	0.37 (±0.17)
Retroactive Familiar	0.34 (±0.16)	0.23 (±0.14)	0.38 (±0.15)	0.36 (±0.18)
Wakeful Rest	0.39 (±0.15)	0.24 (±0.14)	0.39 (±0.15)	0.36 (±0.17)

**Table 2.** Hypotheses of interest, relevant contrasts and inferential statistics. Note, our criterion for ‘conclusive’ evidence was *BF*_10_ or *BF*_01_ exceeding 6. Please note the numbers in the first two rows of this table are identical when rounded to two decimal places.

Hypothesis	Contrast	Data	*BF*_10_	*BF*_01_	Cohen’s *d*	Description
H1: Proactive Novelty Effect	Proactive Novelty (*n* = 120) > Proactive Familiarised (*n* = 120)	Immediate recall	0.13	7.69	−0.02	Conclusive evidence favouring the null hypothesis
		Delayed recall	0.13	7.69	−0.02	Conclusive evidence favouring the null hypothesis
H2: Retroactive Resting Effect	Wakeful Rest (*n* = 168) > Retroactive Familiarised (*n* = 168)	Immediate recall	20.23	0.05	0.33	Conclusive evidence favouring the alternative hypothesis
		Delayed recall	0.25	4.00	0.09	Inconclusive evidence favouring the null hypothesis
H3: Retroactive Novelty Effect	Retroactive Novelty (*n* = 168) > Retroactive Familiarised (*n* = 168)	Immediate recall	0.07	14.29	−0.08	Conclusive evidence favouring the null hypothesis
		Delayed recall	0.06	16.67	−0.12	Conclusive evidence favouring the null hypothesis

We found conclusive evidence for no proactive or retroactive benefit of novelty in both immediate and delayed recalls. Conversely, we found conclusive evidence for the presence of a retroactive benefit of wakeful rest in immediate recall. The evidence for the Retroactive Resting effect in delayed recall was inconclusive, though it favoured the null hypothesis, suggesting that the benefit of post-encoding rest at immediate test may not endure over a 24-h delay.

#### Recognition

The results of the independent dual-process model applied to the 24-h delayed recognition test are summarised in Table 3. We had predicted effects of either novelty or rest would be observable in estimates of recollection, but not in estimates of familiarity. The estimates of recollection (a probability) and familiarity (a *d*’ measure) were not normally distributed, so non-parametric one-tailed Wilcoxon Rank Sum tests were used for statistical (frequentist) inference.

**Table 3.** Median (IQR) estimates of delayed recognition via recollection or familiarity, by group.

Experimental group	Probability of recollection	Familiarity (*d*’)
Proactive Novelty	43.75% (29.36%)	1.42 (1.42)
Proactive Familiarised	50.00% (42.89%)	1.35 (1.30)
Retroactive Novelty	50.00% (43.55%)	1.54 (1.23)
Retroactive Familiarised	50.00% (35.51%)	1.63 (1.56)
Wakeful Rest	50.09% (29.45%)	1.39 (1.48)

There were no significant differences in the probability of recollection for any of the registered hypotheses: between the proactive novelty and proactive familiarised groups (H1: *W* = 6,792.50, *p* = .776); between the wakeful rest and retroactive familiarised groups (H2: *W* = 13,396.50, *p* = .211); nor between the retroactive novelty and retroactive familiarised groups (H3: *W* = 13,893.50, *p* = .597).

Similarly, there were no significant differences in the familiarity estimates for any of these contrasts: between the proactive novelty and proactive familiarised groups (H1: *W* = 7,127.00, *p* = .554); between the wakeful rest and retroactive familiarised groups (H2: *W* = 15,130.00, *p* = .874); nor between the retroactive novelty and retroactive familiarised groups (H3: *W* = 13,817.5, *p* = .630).

In sum, regarding our confirmatory hypotheses, we found no statistically significant effects of novelty or rest in recognition via recollection nor familiarity, when tested 24 h after encoding.

### Exploratory analyses

#### Proactive benefit of rest

The Retroactive Resting benefit that we observed for immediate recall in our confirmatory analyses fits with the predictions of Consolidation theory (Dudai, 2004; Wixted, 2004), namely that effortful tasks performed after encoding disrupt consolidation processes (see ‘Introduction’ section). However, two aspects of our findings are difficult to reconcile with consolidation. Firstly, one might expect consolidation to be disrupted to a greater extent by exploring a novel versus familiar environment, assuming novel exploration is more effortful than familiar exploration, yet there was evidence against this (H3). Perhaps more importantly, Consolidation theory would appear unable to explain any benefit of rest before encoding, and yet our data suggest that recall was better in our wakeful rest group than both proactive exploration groups (see Figure 3).

We therefore conducted exploratory analyses to compare immediate and delayed recall between the wakeful rest group and each of the novel and familiar proactive groups, using two-tailed, unpaired, Bayesian *T*-tests. The results of these tests are displayed in Table 4. For immediate recall, the evidence supported the existence of a Proactive Resting benefit, conclusively relative to the proactive novelty group (the BF almost surpassed our threshold for conclusive evidence relative to the proactive familiarised group). As with the Retroactive Resting benefit, the evidence was inconclusive for delayed recall in both cases.

**Table 4.** Exploratory contrasts relating to the proactive rest effect.

Hypothesis	Contrast	Data	*BF*_10_	*BF*_01_	Cohen’s *d*	Description
Proactive Resting Effect	Wakeful Rest (*n* = 168) > Proactive Novelty (*n* = 120)	Immediate recall	9.02	0.11	0.36	Conclusive evidence favouring the alternative hypothesis
		Delayed recall	1.20	0.83	0.26	Inconclusive evidence favouring the alternative hypothesis
	Wakeful Rest (*n* = 168) > Proactive Familiarised (*n* = 120)	Immediate recall	5.53	0.18	0.34	Inconclusive evidence favouring the alternative hypothesis
		Delayed recall	0.89	1.12	0.24	Inconclusive evidence favouring the null hypothesis

We also tested these exploratory hypotheses for the estimates of recollection and familiarity from the delayed recognition data, using two-tailed Wilcoxon Rank Sum tests. For the recollection estimates, we found significantly increased probability of recollection in the wakeful rest group than the proactive novelty group (*W* = 8,126.50, *p* = .005), while the corresponding comparison against the proactive familiarised group approached significance (*W* = 8,876.00, *p* = .084). We found no significant differences in familiarity estimates in either case (*W* = 9,989.50, *p* = .897 and *W* = 10,094.00, *p* = .985, respectively). Thus, the evidence from the delayed recognition task generally supported the benefit of proactive rest that was found in the immediate recall task, suggesting that the benefit can last 24 h (and that the delayed recall test was simply too noisy to reveal the same effect). However, this benefit is restricted to recollection, with no evidence that it affects familiarity.

#### Role of rehearsal

An alternative explanation often offered for a retroactive benefit of rest is that post-encoding rest simply provides greater opportunity for participants to actively rehearse words in preparation for a later memory test. To reduce the chance of intentional rehearsal, our instructions encouraged participants to simply focus on the current task, for example, for the encoding task, they were instructed to ‘Please just focus on the Pleasantness Task. Your performance in remembering words will not affect your payment’, while for the rest task, they were asked to ‘Take this as an opportunity to rest your mind. There is no need to think about other parts of the experiment’. We did not explicitly instruct participants not to rehearse words because we were concerned that they might actively suppress words in memory and possibly negate potential Novelty or Resting effects (Anderson & Levy, 2009). Nevertheless, because participants did expect the memory tests, we explored the extent to which participants in each group reported deliberately rehearsing words during the retention interval.

**Figure 4.**
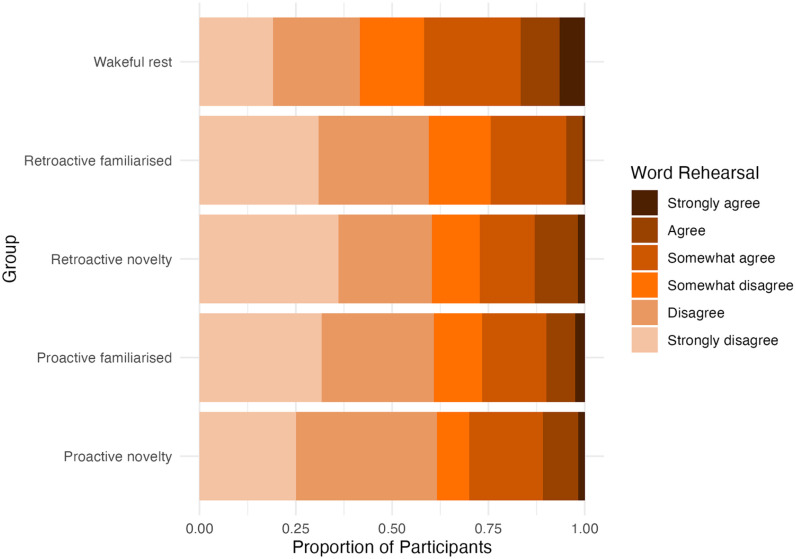
Self-reported levels of word rehearsal during the retention interval on Day 2 across groups.

At the end of session 2, participants were asked to ‘cast their mind back to’ the task that filled the retention interval (for the retroactive groups this was the explore task; for all other groups this was the relaxation task) and report the extent to which they agreed with the statement ‘I deliberately rehearsed the words in my head during this task’ (Six levels: *Strongly agree* to *Strongly disagree*). The results are displayed in Figure 4. A Bayesian ordinal regression model provided conclusive evidence that experimental group predicted word rehearsal responses (*BF*_10_ = 1,793.72, *BF*_01_ < 0.01) better than an intercept-only model, indicating that word rehearsal during the retention interval differed between groups. Figure 4 shows that the wakeful rest group appears to report greater rehearsal than the retroactive novel and retroactive familiar groups, who were engaged with the exploration task during the retention interval. As such, this provides a possible explanation for the retroactive benefit of rest we found. However, Figure 4 shows that the wakeful rest group also appears to report greater rehearsal than the proactive novel and proactive familiar groups. This was unexpected because these participants all experienced the same rest task during the retention interval, so they had the equivalent opportunity to rehearse. Nonetheless, the consequence is that rehearsal may also provide an alternative explanation for the proactive benefit of rest that we found in our exploratory analysis above.

To investigate the extent to which word rehearsal could explain our rest effects, we examined the relationship between the self-reported rehearsal and immediate recalls. We selected pairs of groups relevant to our Retroactive Resting effect (i.e. the retroactive familiarised group and the retroactive novelty group, each compared with the wakeful rest group) and Proactive Resting effect (i.e. the proactive novelty group and the proactive familiarised group, each compared with the wakeful rest group). We asked whether the experimental group still predicted immediate recall after accounting for word rehearsal, by comparing a full model with both the experimental group and word rehearsal as predictors, against a model with only word rehearsal as a predictor. Note that rehearsal was treated as a factor with 6 levels, such that the model could capture any (nonlinear) relationship between rehearsal and memory. For the retroactive familiarised group versus the wakeful rest group, there was conclusive evidence that the experimental group still predicted immediate recall after accounting for word rehearsal (*BF*_10_ = 60.35, *BF*_01_ = 0.02). The evidence was inconclusive for the other pairwise comparisons, but followed the same tendency (i.e. retroactive novelty vs. wakeful rest: *BF*_10_ = 2.05, *BF*_01_ = 0.49; proactive familiarised vs. wakeful rest: *BF*_10_ = 3.10, *BF*_01_ = 0.32; proactive novelty vs. wakeful rest: *BF*_10_ = 2.42, *BF*_01_ = 0.41). Conversely, we asked whether word rehearsal predicted immediate recall when accounting for the experimental group, by comparing a full model with both experimental group and word rehearsal as predictors with an experimental group-only model. Similarly, only the comparison of the retroactive familiarised group with the wakeful rest group produced conclusive evidence, in the direction that rehearsal did not predict recall when the experimental group was accounted for (*BF*_10_ = 0.07, *BF*_01_ = 14.28). The others produced inconclusive evidence following the same tendency (i.e. retroactive novelty vs. wakeful rest: *BF*_10_ = 0.83, *BF*_01_ = 1.20; proactive familiarised vs. wakeful rest: *BF*_10_ = 0.18, *BF*_01_ = 5.66; proactive novelty vs. wakeful rest: *BF*_10_ = 0.30, *BF*_01_ = 3.32).

Finally, we tested whether rehearsal predicted immediate recall within each group separately (by comparing against an intercept-only model). While the evidence was inconclusive for three of the groups (retroactive novel group – *BF*_10_ = 2.50, *BF*_01_ = 0.40; proactive novel group – *BF*_10_ = 0.36, *BF*_01_ = 2.80; proactive familiarised group – *BF*_10_ = 0.44, *BF*_01_ = 2.25), there was actually evidence against the role of rehearsal within the wakeful rest group (*BF*_10_ = 0.11, *BF*_01_ = 9.00) and within the retroactive familiarised group (*BF*_10_ = 0.10, *BF*_01_ = 10.21).

Overall, these results suggest that rehearsal cannot fully explain all the Retroactive and Proactive Resting effects.

## Discussion

This study compared three tasks – virtual exploration of a novel environment, virtual exploration of a familiarised environment and a low-effort attention task of the type previously characterised as ‘wakeful rest’ – that were delivered immediately before and/or after the encoding of a list of unrelated words. Comparisons of subsequent memory for the words between groups who did different combinations of these tasks allowed us to explore the proactive and retroactive effects of novelty and rest, as two potential interventions for improving memory.

Contrary to some previous studies, BFs for our first and third hypotheses regarding the beneficial effect of novelty provided evidence that novel exploration does not proactively or retroactively benefit free recall, relative to familiar exploration, neither in an immediate test, nor after a 24-h delay. In fact, novel exploration appeared to impair memory, given that immediate recall before/after novel exploration was generally worse than in a ‘baseline’ test on the previous day; conclusively so for the retroactive novelty group (see Supplemental Material). Likewise, there was no evidence for a proactive or retroactive benefit of novelty on a delayed test of recognition memory, regardless of whether memory was assessed by estimates of recollection or familiarity (using an independent, dual-process model). Thus, we did not replicate previous findings of a proactive (Abrahan et al., 2020; Schomaker & Wittmann, 2021; Schomaker et al., 2014, 2022) or retroactive (Ballarini et al., 2013; Ramirez Butavand et al., 2020) benefit of novel experiences in human memory. Instead, we replicated other studies that did not find a novelty effect (Biel & Bunzeck, 2019; Biel et al., 2020; McClay & Dunsmoor, 2018; Quent and Henson, 2022; Sazma et al., 2023). Our evidence against both retroactive and proactive effects of novelty questions BTT, which was developed from effects of novel experiences in animals, at least with the type of novelty and memory tests used here in humans.

Consistent with other previous studies (Craig et al., 2014; Dewar et al., 2012, 2014; King & Nicosia, 2022) BFs for our second hypothesis regarding the beneficial effect of rest provided evidence that a period of wakeful rest after encoding does benefit immediate free recall, relative to exploration of a familiar environment. Evidence that this benefit of rest lasted 24 h was inconclusive, however, when memory was assessed with recall and recollection during recognition.

One explanation for this immediate benefit of post-encoding rest – or at least a task with minimal cognitive demands – is that participants find it easier to actively rehearse the words. We told participants to focus only on the tasks at hand, but they may have rehearsed words during the wakeful rest period, particularly since they were aware (from Day 1) that their memory for the words would be tested. Indeed, debriefing revealed that participants said they were more likely to deliberately rehearse words in the wakeful rest group than any of the other groups. Nonetheless, there was evidence that these rehearsal ratings did not relate to memory within the wakeful rest group (BFs conclusively favoured the null), and the evidence continued to favour the detrimental effect of post-encoding exploration even when adjusting for rehearsal ratings; conclusively so for the retroactive familiar group relative to wakeful rest group. Thus, increased rehearsal in the rest group does not fully explain our pattern of results.

Further to this, there are other studies finding a benefit of rest that cannot be explained by active rehearsal. For example, some studies have used material that is perhaps more difficult to rehearse, such as scrambled words (Dewar et al., 2014) and motor skill tasks (Humiston & Wamsley, 2018; Mylonas et al., 2024; Wang et al., 2021). Others employed incidental encoding and unexpected memory tests (Millar & Balota, 2022), meaning participants had no incentive to rehearse. Even when participants were instructed to forget a list of items as part of a directed forgetting paradigm, and thus incentivised against rehearsing items, there was greater recall for these items after passive rest than after an active distractor task (Schlichting & Bäuml, 2017). Together, these findings suggest that, whilst we cannot completely rule out intentional rehearsal as contributing to the benefits of rest observed in our study, there also seems to be a mechanism by which wakeful rest benefits memory without rehearsal.

An alternative explanation for the retroactive benefit of rest is provided by Consolidation theory, if effortful tasks like exploration are assumed to impair consolidation (Dudai, 2004; Wixted, 2004). Note that this consolidation would need to be very rapid (occurring over 5 min in the present design), and any absence of a rest benefit after a delay would have to be attributed to reduced sensitivity of delayed tests. Yet another account that fits better with the lack of retroactive effect after a delay comes from Temporal Context or Item-Context Binding models (Howard & Kahana, 2002; Yonelinas et al., 2019). These models suggest that memory is impaired in the retroactive exploration groups because their internal mental representation of context was altered during the retention interval to a greater extent by the exploration task than by wakeful rest (Delaney et al., 2010; DuBrow et al., 2017). This would mean that the context present at the start of recall overlapped less with that during encoding, and therefore served as a less effective retrieval cue. These models do not make claims about consolidation processes, so they do not predict that delay per se is important for the effects.

However, both Consolidation theory and Temporal Context models would appear unable to explain a further finding of our study, based on exploratory analysis of the wakeful rest group against the two proactive exploration groups: BFs suggested superior immediate recall in the rest group than both proactive groups; conclusively so relative to the novel exploration group. BFs for any differences in delayed recall were inconclusive, though interestingly, there was evidence that recollection (but not familiarity) was more likely after rest in the delayed recognition task. Thus, wakeful rest prior to encoding also benefits memory, suggesting that rest has proactive as well as retroactive benefits. Since consolidation can only occur after encoding, and a detrimental mental context shift would have to occur during the retention interval, this proactive benefit of rest is difficult for these theories to explain.

One explanation for the proactive benefit of rest is that exploration caused fatigue, which impaired participants’ ability to encode the words. Indeed, the ratings of arousal that we acquired at debriefing suggested (perhaps unsurprisingly) that the exploration tasks were more arousing than the wakeful rest task. Nonetheless, after adjusting for arousal ratings (see Supplemental Material), evidence remained that immediate recall was better following rest than following exploration of either a familiar or novel environment. Another index of fatigue could be response times (RTs) during the encoding task. However, evidence showed that encoding RTs did not differ between rest and proactive exploration groups (see Supplemental Material), again providing no support for the fatigue hypothesis.

An alternative possible explanation of our symmetrical effects of both retroactive and proactive benefits of rest comes from Temporal Distinctiveness theory (Bjork & Whitten, 1974; Brown & Lewandowsky, 2010; Brown et al., 2007; Glenberg & Swanson, 1986). This theory claims that memory performance is determined by competition between target material and interfering material at retrieval. When target and interfering material are encoded relatively close together in time, there is increased competition, impairing retrieval. As a result, wakeful rest may be beneficial because it reduces interference by temporally segregating target material from other potentially interfering material. Furthermore, memory performance is predicted to depend on the ratio of the interval between presentation of different material and the interval between that material and test (i.e. retention interval). This ‘ratio rule’ could potentially explain why any benefits of rest before/after encoding appeared greater on the immediate test than on the delayed test. A potential weakness of this explanation is that it requires that information retrieved about the exploration tasks competes with that retrieved about the target words, despite the different nature of this information (or more precisely, that information from the exploration tasks competes to a greater extent than any information presented/generated by participants during the resting task).

Indeed, the benefit of pre-encoding rest on memory has been shown before. For example, in two experiments Ecker et al. (2015), had participants encode three separate word lists and probed recall of the second list. Across four conditions, they manipulated the duration of a period of wakeful rest between the encoding of each list (i.e. the pre-/post-encoding period for List 2). The time between List 2 encoding and recall was matched across conditions. They found consistent support for a main effect of pre-encoding rest, whereby List 2 recall was improved when the pre-encoding rest period was longer. They also found, less consistently, a comparable main effect of post-encoding rest: Only in the second experiment, where the long rest period was halved so that the long and short rest periods were more similar in duration. In another study, they replicated similar experimental findings and showed the data could be explained by a computational implementation of Temporal Distinctiveness theory (Brown et al., 2007) without the need for an additional component representing consolidation mechanisms (Ecker et al., 2015).

Temporal distinctiveness is easy to apply to Ecker et al.’s studies because the interfering lists contained similar materials (words), which we know from many studies of verbal learning and memory will interfere at retrieval (e.g. Underwood, 1957). More similar to our study is a study by McGhee et al. (2020), who found evidence that both pre- and post-encoding rests benefited recall in patients with amnesia. They found that giving patients an effortful task immediately before and after learning impaired recall of a short story, relative to wakeful rest before and after learning. The effortful task was a visual spot-the-difference task, which meant, like our study, the target material (verbally presented prose) was a different format from the interfering material (visual images). However, unlike our study, they also found that wakeful rest both before and after encoding was no better than wakeful rest on one side of encoding and an effortful task on the other (regardless of order). This is not consistent with Temporal Distinctiveness, nor with Consolidation theory. Thus, while aspects of the present findings and those of previous studies are consistent with Temporal Distinctiveness theory, there are other aspects, such as the nature of interference between different types of material and the relative roles of rest before versus after encoding, that require further investigation.

### Caveats

There are, of course, several caveats with our interpretations. Foremost is the possibility that our exploration of a novel relative to familiar environment was not novel enough to observe the type of novelty benefits seen when, for example, a rodent is put in a completely new cage (Ballarini et al., 2009; Justel et al., 2021; Moncada & Viola, 2007), or when school children experience a novel lesson (Ballarini et al., 2013; Ramirez Butavand et al., 2020). Along this vein, studies with rodents are likely to involve considerably greater modulation of stress and arousal than human studies, which could be responsible for the effect (King & Williams, 2009). Indeed, we did not find any evidence of intervention-related differences in arousal, in terms of self-reported arousal ratings or RTs during the encoding task (see Supplemental Material), which could explain why we did not find a Novelty effect (if the Novelty effect is mediated by arousal). Nonetheless, our manipulation was based on very similar manipulations by Schomaker et al. (2022), who did find memory benefits of (proactive) novel exploration for participants of a similar age as the current sample. One possibility is that our sample of participants contained a larger proportion of computer gamers (particularly given our online recruitment), for whom these types of exploration tasks may be much less novel or arousing. Nonetheless, despite the relatively smaller sub-group of our participants who were not gamers, BFs still provided evidence against the possibility that the size of the novelty effect depends on whether a participant is a gamer (see Supplemental Material). Furthermore, Schomaker et al. (2014) found a novelty effect when exploration of a novel and familiar virtual environment was compared within-participant, suggesting it is the novelty of the virtual environment and not the task that is important. One factor affecting this could be exploration duration. We used identical VEs to Schomaker et al. (2022), but our participants explored for longer (5 min vs. 3 min). Schomaker et al. (2014) used 5 min of exploration, but this was with different environments, which may have had more space to roam and used headset VR, which may have felt more novel. If our participants were relatively more familiar with the environment by the end of the task, this could have reduced the novelty of the experience. Nevertheless, insufficient novelty does not explain Quent and Henson’s (2022) evidence against a retroactive novelty effect because none of their participants had ever experienced immersive VR before, so they should have found the intervention to be highly novel. Thus, we do not see strong reasons why our study induced any less novelty than previous studies using virtual reality exploration, though we accept that this level of novelty could still be much smaller than in animal studies or some other real-world human studies.

Another potential caveat concerns our use of online testing. This was practically necessary to obtain sufficient power to test all our hypotheses across groups of participants. Nonetheless, the quality of the data produced is likely to be lower than in laboratory settings where an experimenter can supervise participants directly. It is possible that some of our participants disobeyed our instructions, and for example, multitasked during the experiment, which might attenuate any true differences between groups. Nonetheless, we took considerable precautions, particularly in relation to the wakeful rest task. Rather than having complete wakeful rest, which would have been impossible to supervise, we included a very undemanding change detection task, based on one that has been used in previous wakeful rest studies (Dewar et al., 2010). We used online performance checks so that if a participant lost focus and did not detect enough of the events, navigated away from the computer screen, or responded continuously/randomly throughout the task, they were immediately terminated from the experiment. It is possible that some participants still managed to multitask and maintain performance on the task to bypass the performance checks, but given the stringent criteria (70% performance threshold), we feel that most participants in the final sample would have been adequately engaged and adhered to instructions. Furthermore, our offline data exclusion rates were relatively low for online research (Thomas & Clifford, 2017), indicating high standards of data quality. We suspect that sufficient performance-based attention checks and preregistered data exclusion criteria help ensure that online experiments provide behavioural data of sufficient quality, but it remains possible that the use of online testing could have reduced the sensitivity of our paradigm.

Finally, it is possible that our measures of delayed memory performance (after 24 h) were not sensitive enough. Delayed recall performance was close to the floor and may have been more severely affected by proactive interference from the Baseline 1 word list than immediate recall. We also included a delayed recognition test, but our estimates of recollection and familiarity from the independent dual-process model could have been noisy because we had far fewer recognition trials than typical studies of recognition memory (Yonelinas & Parks, 2007) – given that our word lists had to be short enough to also ensure that recall was off floor. Thus, our results regarding delayed memory performance, from both our recall and recognition tests, should be treated with caution.

## Conclusion

In summary, the present study provides evidence against the notion that exploring a novel virtual environment improves memory for unrelated material occurring shortly before or afterwards. It also provides evidence supporting the proactive and retroactive beneficial effects of wakeful rest, compared with more effortful tasks, at least when memory is tested shortly afterwards. The finding of a proactive benefit of rest is more consistent with Temporal Distinctiveness theory than Consolidation theory, though Temporal Distinctiveness theory may need elaboration to account for interference between the retrieval of quite different types of information. Moreover, wakeful rest appears to be a useful intervention for improving memory performance, with possible applications for improving memory in people with memory problems.

## Supplemental Material

sj-docx-1-qjp-10.1177_17470218251346156 – Supplemental material for Proactive and retroactive effects of novelty and rest on memory Supplemental material, sj-docx-1-qjp-10.1177_17470218251346156 for Proactive and retroactive effects of novelty and rest on memory by Sumaiyah Raza, Judith Schomaker, Jörn Alexander Quent, Michael C Anderson and Richard N Henson in Quarterly Journal of Experimental Psychology
