# Supplementary material for: Proactive and retroactive effects of novelty and rest on memory
Source: Q J Exp Psychol (Hove). 2025 May 21;79(2):267–84. doi: 10.1177/17470218251346156 (PMC12796013; doi:10.1177/17470218251346156)
Supplement: sj-docx-1-qjp-10.1177_17470218251346156 – Supplemental material for Proactive and retroactive effects of novelty and rest on memory [file sj-docx-1-qjp-10.1177_17470218251346156.docx]

## Supplementary Materials for:

PROACTIVE AND RETROACTIVE EFFECTS OF NOVELTY AND REST ON MEMORY.

*Sumaiyah Raza^1^*

*Judith Schomaker^3,4^*

*Jörn Alexander Quent^5,6,7^*

*Michael C. Anderson^1^*

*Richard N. Henson^1,2^*

*^1^MRC Cognition & Brain Science Unit, University of Cambridge, Cambridge, UK.*

*^2^Department of Psychiatry, University of Cambridge, Cambridge, UK.*

*^3^Department Health, Medical and Neuropsychology, Leiden University, Leiden, The Netherlands.*

*^4^Department for Social and Behavioural Sciences, Leiden University, Leiden, The Netherlands.*

*^5^Institute of Science and Technology for Brain-Inspired Intelligence, Fudan University, Shanghai, China.*

*^6^Key Laboratory of Computational Neuroscience and Brain-Inspired Intelligence (Fudan University), Ministry of Education, China.*

*^7^MOE Frontiers Center for Brain Science, Fudan University, Shanghai, China.*

### Word Stimuli

The word stimuli are shown in Table S1. Kruskal-Wallis tests indicated that there were no significant differences between any of the lists (both the original and final stimulus sets) in terms of the following psycholinguistics characteristics: familiarity rating, imageability rating, Kucera-Francis number of categories, Kucera-Francis number of samples, Kucera-Francis written frequency, number of letters, number of syllables, number of phonemes, Colorado Norm meaningfulness ratings, and concreteness ratings (all p-values >.583).

*Table S1. Word stimuli. Lists A-C were used in each encoding phase and the list of lures was used in the recognition test. The order of lists A-C was counterbalanced such that equal numbers of participants in each group experienced the lists in each of the 6 possible orders: ABC, ACB, BAC, BCA, CAB, CBA. The lures were constant across all participants. As explained in the main paper, the stimulus set was altered after the first 32 participants because of feedback regarding the suitability of some of the words for non-native English speakers (our registered inclusion criteria). The words marked with an asterisk in the table were originally: (from A) bauble, sapling, (from B) gazelle, janitor, stairway, trout, (from C) beaker, matron, wicket, and (from Lures) skylark.*

| Set | List | Words |
| --- | --- | --- |
| Final stimulus set | A | ANCHOR ATHLETE BISCUIT* BUBBLE DOLPHIN ENZYME* GERM GRAVE LUNAR MOLE MOUSTACHE PANTHER SKULL SLEEVE SNORKEL SPACE |
|  | B | ANKLE ARTERY* BLADDER CALF CANVAS* CIRCUIT FEAST GLACIER HARDWARE LATTICE OLIVE PARCEL SILK* SLEDGE STALLION* THIGH |
|  | C | ACROBAT AGENDA CONCRETE FLUTE FROST HOLLOW* HORMONE MAGICIAN OYSTER* POSTER PRAWN RODENT ROOSTER SAUCER* SWORD TONGUE |
|  | Lures | CAVE DUCK DWARF FILTER JEWEL KITTEN MOLECULE MONKEY MUSHROOM PANDA* PODIUM POPE SOCKET SPATULA TATTOO WOMB |
|  |  |  |

### Power Analysis

To determine the ‘power’ of our design, we performed 10,000 simulations of two one-tailed, unpaired Bayesian T-tests (code adapted from <https://github.com/LevanBokeria/cbu_bayesian_sequential_designs/tree/multiple_stopping_rules>

), for each of our hypotheses with effect sizes (1) taken from previous literature for H1, or (2) set to zero for H0. For Hypothesis 1 (Proactive Novelty effect), we set Cohen’s d=0.44, based on half that of Schomaker et al. (2014). We halved this effect size to account for potential effect size inflation, e.g. due to publication bias, because the estimate is based on only one study. For Hypothesis 2 (Retroactive Resting effect), we set Cohen’s d=0.38, based on a meta-analysis of 10 studies investigating post-encoding rest (Humiston et al., 2019).

Our simulations (see Table S2) demonstrate that, with a maximum N of 168 per experimental group, our study was well-powered (i.e. ~80% power) to detect both of our main effects of interest if they both exist (Novelty effect/Resting effect). If neither effect exists, our study had a moderate chance of finding conclusive evidence for the null hypothesis in both cases (i.e. ~60% power). Our study had a ~85-95% probability of finding conclusive evidence for H1 or H0 for at least one effect (i.e. the sum of the probability of conclusive and partially conclusive evidence). Across all combinations of H1 and H0, our study had a low chance of producing misleading evidence (i.e. < 4% false positive rate). Furthermore, we our simulations suggested we were likely to reach conclusive evidence (i.e. terminate our experiment) before recruiting our maximum N of 168 per group. For example, to obtain conclusive evidence of both H1s when both are true, the median number of participants required across all our simulations was 108 per group (total N=540). Similarly, to obtain conclusive evidence of both H0s when neither effect exists, the median number of participants required was 120 per group (total N=600).

*Table S2. Bayesian power analysis. Percentage of simulations providing evidence of various types for our two main effects of interest (corresponding to the probability of obtaining evidence of various types in our study).*

|  | Both effects exist.  (Cohen’s d=0.38 & 0.44) | Only the Novelty effect exists.  (Cohen’s d=0.0 & 0.44) | Only the Resting effect exists.  (Cohen’s d=0.38 & 0.0) | Neither effect exists.  (Cohen’s d=0 & 0) |
| --- | --- | --- | --- | --- |
| Conclusive evidence for both hypotheses. ^1^ | 79.71% | 73.09% | 65.05% | 59.86% |
| Partially conclusive evidence. ^2^ | 16.54% | 21.89% | 25.43% | 26.53% |
| Inconclusive evidence for both hypotheses. ^3^ | 1.28% | 5.78% | 2.39% | 10.07% |
| Misleading evidence for either hypothesis. ^4^ | 2.47% | 2.63% | 3.74% | 3.54% |

^1^ Conclusive evidence refers to a BF exceeding criterion that is not misleading, e.g., in the case that both effects exist (column 1 of Table) this would equal BF10>6 for both effects; in the case that only the Novelty effect exists (column 2 of Table) this would equal BF10>6 for the Novelty effect and BF01>6 for the Resting effect).

^2^ Partially conclusive evidence refers to a not-misleading BF exceeding criterion for one effect, and not exceeding criterion for the other effect, e.g., in that case that only a Resting effect exists (column 3 of Table), this could be a BF10>6 for the Resting effect and a BF not exceeding criterion for the Novelty effect or a BF01>6 for the Novelty effect and a BF not exceeding criterion for the Resting effect.

^3^ Inconclusive evidence refers to a case where BFs for both effects do not exceed criterion.

^4^ Misleading evidence refers to cases where one or both BFs exceeded criterion for the incorrect hypotheses compared with the effects actually in existence, e.g., where neither effect exists (column 4 of Table) this could be BF10>6 for one or both effects.

### Complete Recall Data (incl. Baselines)


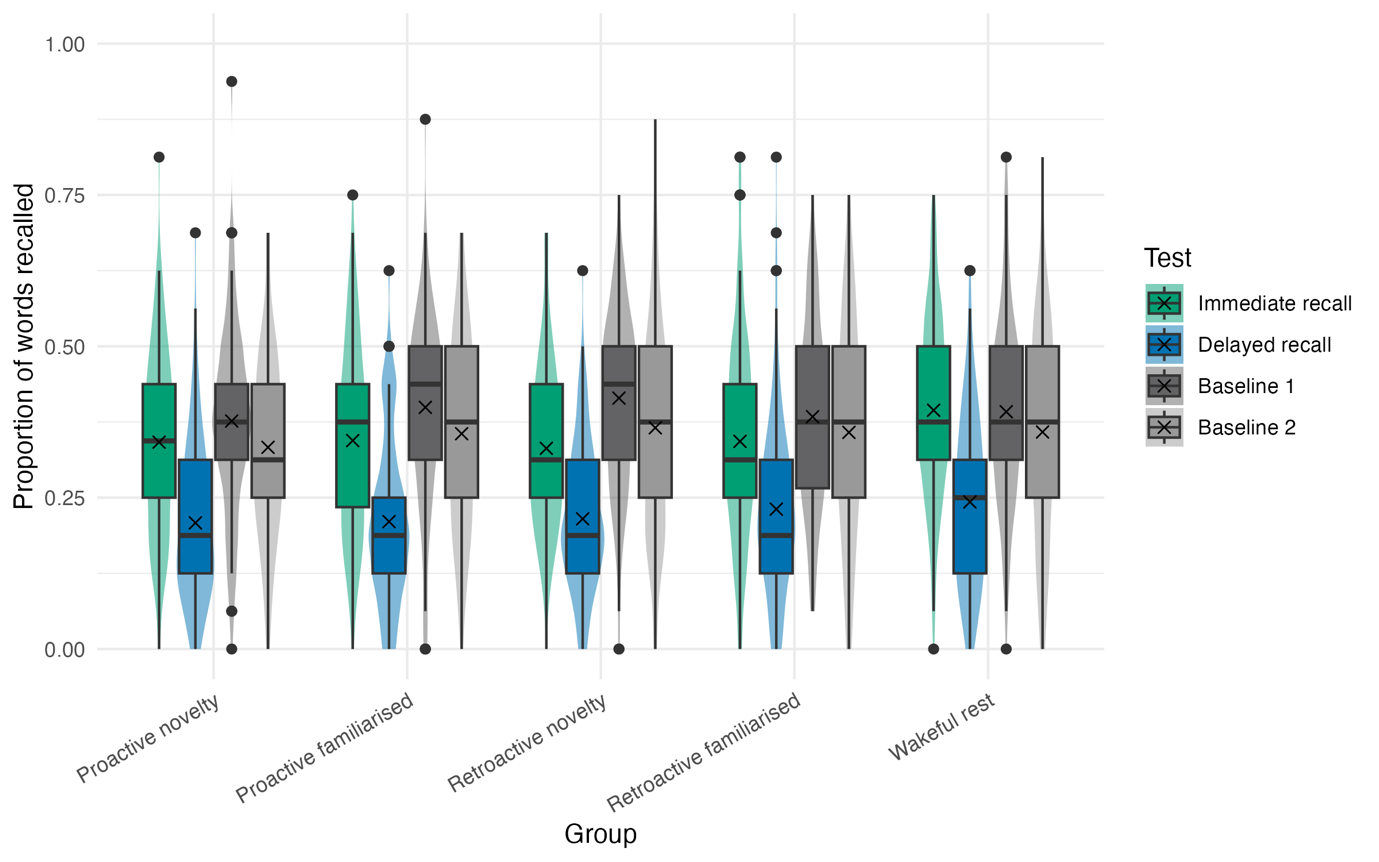


*Figure S1. Distribution of recall scores for each type of recall test, by group. Group means for each test are represented with Xs. Note, dots represent recall scores falling outside 1.5x the IQR of each group and test type separately. These datapoints are not outliers in terms of the pooled immediate recall scores upon which outlier removal was performed (see Outlier Removal section).*

### Baseline correction


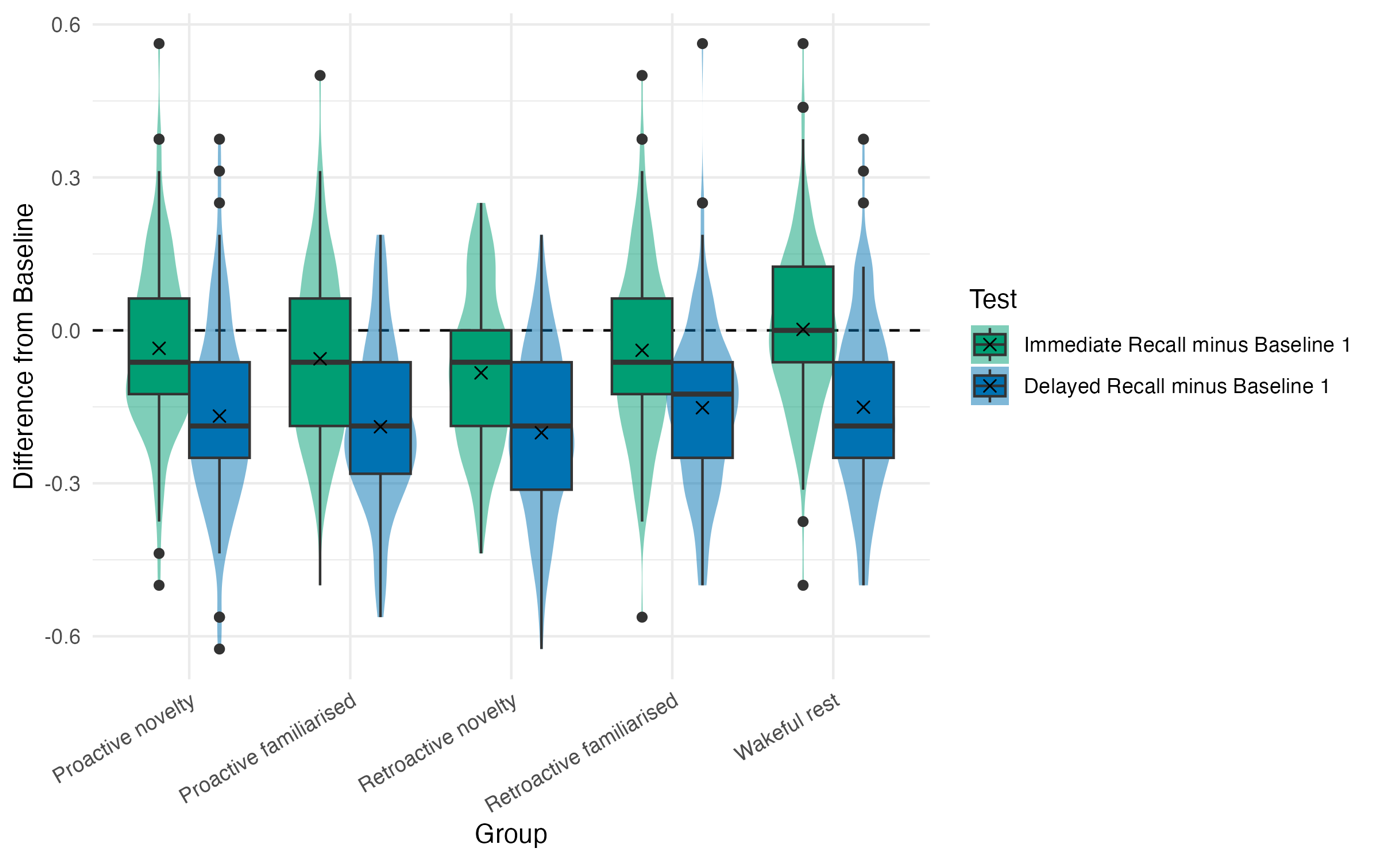


*Figure S2. Difference from baseline for immediate and delayed recall data across groups (i.e., intervention-related scores minus Baseline 1 scores). Mean difference scores are represented with Xs; dots represent outliers in terms of the spread of difference scores – these were not removed because they were not deemed outliers in terms of the pooled immediate recall scores upon which outlier removal was performed (see Outlier Removal section).*

As registered in Stage 1, we acquired ‘baseline’ recall scores on Day 1 and Day 3 for use in removing some of the variance due to individual differences in memory ability. However, the Pearson’s correlation between Baseline 1 and Baseline 2 scores was less than 0.5 (r=0.42), so we chose to use only the Baseline 1 test as our measure of baseline performance, because there were no procedural differences between groups by this point in the experiment. Figure S2 displays the difference from baseline across groups.

We first used this baseline performance measure to test whether intervention-related immediate recall performance differed from participants’ own typical performance. For each experimental group, we conducted a Bayesian, two-tailed, paired T-test comparing participant’s Baseline 1 score as measured on Day 1, before any intervention, with their immediate recall score on Day 2, after the intervention.

The baseline test procedure was comparable to the wakeful rest intervention, so the two types of scores were not expected to differ for participants in the wakeful rest group. Indeed, we found conclusive evidence that intervention-related recall did not differ from Baseline 1 recall in the wakeful rest group (BF10=0.09, BF01=11.11, d=0.01). By contrast, there was conclusive evidence that the retroactive novelty group (BF10>1000, BF01<0.01, d=-0.60), proactive familiarised group (BF10=62.00, BF01=0.02, d=-0.37) and the retroactive familiarised group (BF10=6.81, BF01=0.15, d=-0.25) performed worse than baseline after the intervention. Finally, there was inconclusive evidence that the proactive novelty group (BF10=1.28, BF01=0.78, d=-0.21) performed worse than baseline. It is worth noting that despite there being no differences in experience between groups by the Baseline 1 test, participants in the proactive novelty group tended to have worse performance in the Baseline 1 test than other groups; the reason for which is unclear. This is probably why the evidence was inconclusive regarding the proactive novelty group’s difference from baseline. In sum, these results suggest that exploration tasks proactively and retroactively impair memory.

We also regressed intervention-related immediate recall scores against Baseline 1 scores (concatenated across all groups), to attempt to remove the effect of individual differences in memory performance. We chose this method of “baseline-correction” over subtracting each person’s scores because it is generally less sensitive to noise (Van Breukelen, 2006). We repeated the contrasts from the main paper using this baseline-corrected data - the results are listed in Table S3. When repeating the pairwise comparisons across groups that were used in our confirmatory and exploratory analyses in the main paper, the Bayes Factors for these baseline-corrected scores were similar, except that: (1) evidence for the Proactive Novelty effect reduced from conclusively favouring the null, to be inconclusive for both immediate and delayed baseline-adjusted recall (although the BFs were very close to 6 and still favoured the null), and (2) evidence for the Proactive Resting effect, when compared against the proactive familiarised group, strengthened from inconclusive to strong in favour of the alternative hypothesis.

As mentioned, this analysis should be interpreted with caution because baseline-correction may introduce additional noise when the baseline scores themselves are noisy (i.e., if measurement noise exceeds individual differences in memory). Nevertheless, this analysis mirrors the main paper, supporting the existence of a bidirectional Resting effect and the absence of a Novelty effect.

*Table S3. All confirmatory and exploratory contrasts with baseline-corrected recall data. Note that H1-3 were one-tailed tests, and the exploratory Proactive Resting effect tests were two-tailed to make each comparable with the main analysis.*

| Hypothesis | Contrasts | Data | BF10 | BF01 | Cohen’s d | Description |
| --- | --- | --- | --- | --- | --- | --- |
| H1: Proactive Novelty Effect | Proactive Novelty (n=119) > Proactive Familiarised (n=119) | Baseline-corrected immediate recall | 0.20 | 5.00 | 0.05 | Inconclusive evidence in favour of the null hypothesis. |
|  |  | Baseline-corrected delayed recall | 0.19 | 5.26 | 0.04 | Inconclusive evidence in favour of the null hypothesis. |
| H2: Retroactive Resting Effect | Wakeful Rest (n=166) > Retroactive Familiarised (n= 166) | Baseline-corrected immediate recall | 17.86 | 0.06 | 0.33 | Conclusive evidence in favour of the alternative hypothesis. |
|  |  | Baseline-corrected delayed recall | 0.19 | 5.26 | 0.05 | Inconclusive evidence in favour of the null hypothesis. |
| H3: Retroactive Novelty Effect | Retroactive Novelty (n=165) > Retroactive Familiarised (n=166) | Baseline-corrected immediate recall | 0.05 | 20.00 | -0.20 | Conclusive evidence in favour of the null hypothesis. |
|  |  | Baseline-corrected delayed recall | 0.04 | 25.00 | -0.23 | Conclusive evidence in favour of the null hypothesis. |
| Exploratory: Proactive Resting Effect | Wakeful Rest (n=166) > Proactive Novelty (n=119) | Baseline-corrected immediate recall | 6.32 | 0.16 | 0.34 | Conclusive evidence in favour of the alternative hypothesis. |
|  |  | Baseline-corrected delayed recall | 0.66 | 1.52 | 0.22 | Inconclusive evidence in favour of the null hypothesis. |
|  | Wakeful Rest (n=166) > Proactive Familiarised (n=166) | Baseline-corrected immediate recall | 23.54 | 0.04 | 0.40 | Conclusive evidence in favour of the alternative hypothesis. |
|  |  | Baseline-corrected delayed recall | 1.36 | 0.74 | 0.27 | Inconclusive evidence in favour of the alternative hypothesis. |

### Group outliers

In the main analysis, outliers were determined by pooling the recall scores across groups so as not to bias the results. We also tried excluding outliers separately for each group, for both immediate and delayed recall data, to check that this did not alter the findings. In this case, outliers were scores falling outside of 1.5xIQR calculated for each group and recall test (immediate/delayed) separately. This resulted in 8 participants being removed for immediate recall (proactive novelty – 1, proactive familiarised – 1, retroactive familiar – 5, wakeful rest – 1) and 12 participants being removed for delayed recall (proactive novelty – 1, proactive familiarised – 4, retroactive novelty – 1, retroactive familiarised – 4, wakeful rest – 2). The results remained the same (see Table S4), in terms of conclusive evidence, except for: (1) evidence for the Proactive Novelty effect conclusively supported the null hypothesis for delayed recall in the main paper, but reduced to inconclusive here (although the BF was very close to 6 and still favoured the null), and (2) evidence for the Proactive Resting effect, relative to the proactive familiarised group, for immediate recall was inconclusive in the main paper, but conclusively favoured the alternative hypothesis here. Generally, this analysis still supports the existence of a bidirectional Resting effect and the absence of a Novelty effect.

*Table S4. Inferential statistics for all contrasts with outliers removed from each group separately.*

| Hypothesis | Contrast | Data | BF10 | BF01 | Cohen’s d | Description |
| --- | --- | --- | --- | --- | --- | --- |
| H1: Proactive Novelty Effect | Proactive novelty > Proactive Familiar | Immediate recall | 0.13 | 7.69 | -0.02 | Conclusive evidence in favour of the null hypothesis. |
|  |  | Delayed recall | 0.19 | 5.26 | 0.04 | Inconclusive evidence in favour of the null hypothesis. |
| H2: Retroactive Resting Effect | Wakeful Rest > Retroactive Familiar | Immediate recall | 1056.01 | <0.01 | 0.47 | Conclusive evidence in favour of the alternative hypothesis. |
|  |  | Delayed recall | 0.61 | 1.64 | 0.16 | Inconclusive evidence in favour of the null hypothesis. |
| H3: Retroactive Novelty Effect | Retroactive Novelty > Retroactive Familiar | Immediate recall | 0.13 | 7.69 | 0.01 | Conclusive evidence in favour of the null hypothesis. |
|  |  | Delayed recall | 0.08 | 12.50 | -0.06 | Conclusive evidence in favour of the null hypothesis. |
| Exploratory: Proactive Resting Effect | Wakeful Rest > Proactive Novel | Immediate recall | 33.81 | 0.03 | 0.41 | Conclusive evidence in favour of the alternative hypothesis. |
|  |  | Delayed recall | 1.69 | 0.59 | 0.28 | Inconclusive evidence in favour of the alternative hypothesis. |
|  | Wakeful Rest > Proactive Familiarised | Immediate recall | 16.28 | 0.06 | 0.38 | Conclusive evidence in favour of the alternative hypothesis. |
|  |  | Delayed recall | 3.70 | 0.27 | 0.32 | Inconclusive evidence in favour of the alternative hypothesis. |

### Normalised Data

Upon visual inspection, the immediate recall data appeared to be normally distributed. The delayed recall data may have been skewed slightly by floor effects due to poor recall performance across all groups, however, the skew did not appear to be large. To ensure any possible violations of normality did not influence the results, we repeated the contrasts from the main paper on normalised data. We used the R package ‘BestNormalise’ to select an appropriate transformation, which was a standardised square root transformation for both immediate and delayed recall. This analysis yielded no differences in the pattern of conclusive evidence.

### Arousal

One explanation often put forth to explain proactive effects of an intervention on memory is that the intervention may affect arousal: either improving attentiveness and response times on subsequent encoding tasks via increased arousal, thereby benefitting memory; or impairing attentiveness and slowing response times through reduced arousal (e.g., tiredness), thereby harming memory. It is possible for example that the proactive exploration tasks caused fatigue that reduced arousal during the subsequent study phase, and this led to a memory decrement in these groups. To address this, we explored self-reported levels of arousal for the exploration and rest tasks experienced on Day 2 prior to encoding, as well as response times during the encoding task.

In terms of self-reported arousal prior to encoding, participants were given the definition “arousal refers to your general level of alertness, excitement or energy” and had 6 response levels (‘Very aroused’ to ‘Very calm’). We selected only the three groups that differed in terms of pre-encoding experiences (proactive novel, proactive familiar and wakeful rest groups) experienced on Day 2. These data are displayed in Figure S3.


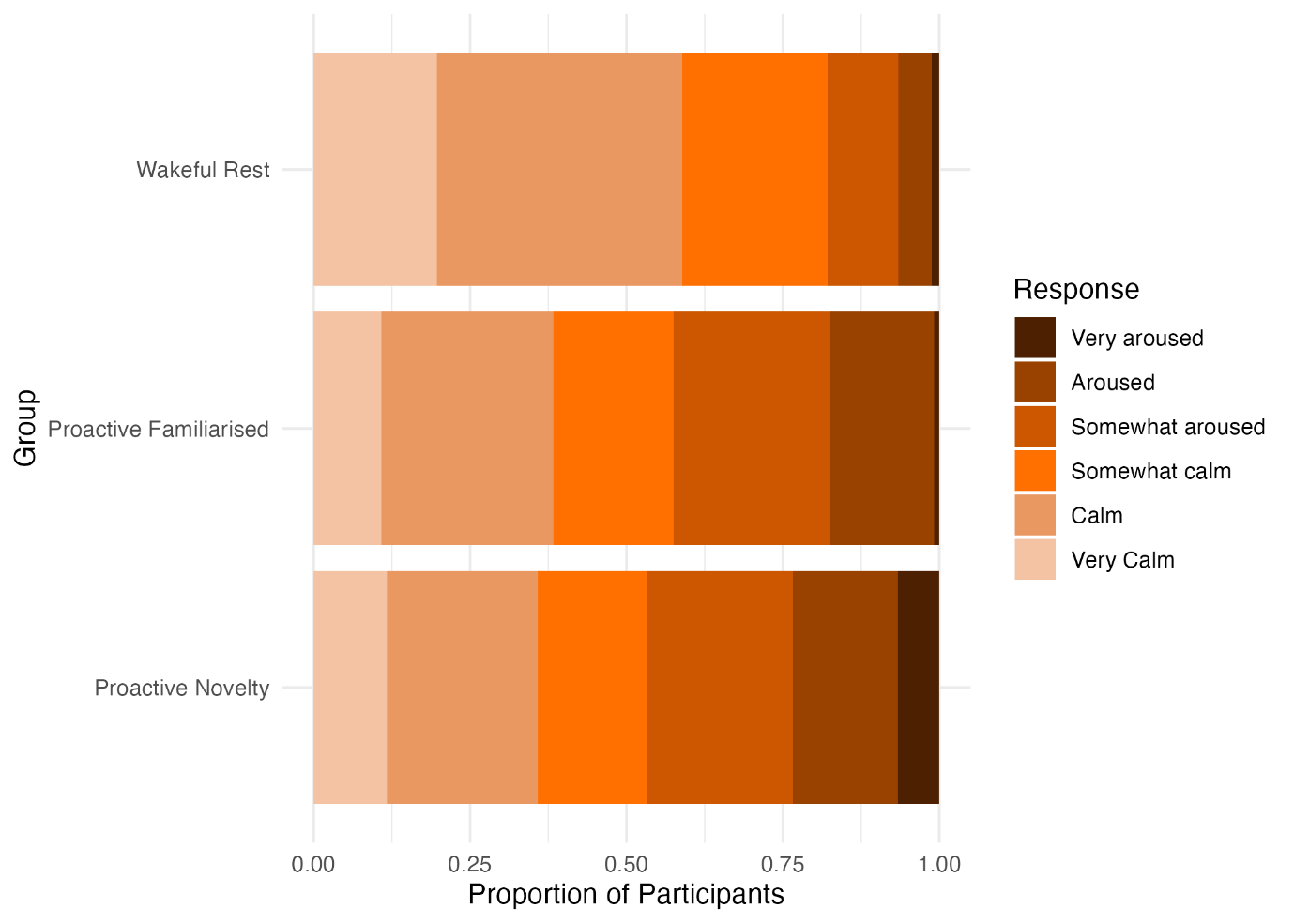


*Figure S3. Self-reported arousal in the pre-encoding task*

A Bayesian ordinal regression model provided conclusive evidence that the three types of experimental group predicted arousal ratings better than the intercept-only model (BF10>1000, BF01<0.01), indicating the level of self-rated arousal pre-encoding differed between groups. Repeating this model comparison with pairs of groups suggested that the proactive groups did not differ from each other – the evidence was inconclusive but favoured the null hypothesis (BF10=0.83, BF01=1.20). Instead, there was conclusive evidence that the wakeful rest group differed from both the proactive groups in terms of arousal ratings (both BF10s>1000).

Given that we found a Proactive Resting effect but no Novelty effect, if arousal were the cause of the group differences in memory, it would be that exploration was so arousing that it caused fatigue for the subsequent study phase, impairing memory (rather than any benefits of such arousal carrying over to the study phase and benefitting memory). To test this, we selected pairs of groups relevant to our Proactive Resting effect (i.e., the proactive novelty group and the proactive familiarised group, each compared with the wakeful rest group). As we did for our analysis of word rehearsal data in the main paper, we compared a full model with both experimental group and arousal as predictors, against a model with only arousal as a predictor, with arousal treated as a factor (with 6 levels) to capture potential nonlinear relationships. For the proactive novelty group compared with the wakeful rest group, experimental group still predicted immediate recall when arousal was accounted for – conclusively so for the comparison between the proactive novelty group and wakeful rest group (BF10=10.32, BF01=0.10) and almost conclusively for the comparison between the proactive familiarised group and wakeful rest group (BF10=5.82, BF01=0.17). By contrast, when experimental group was accounted for, there was conclusive evidence against arousal as a predictor of immediate recall (proactive novelty versus wakeful rest - BF10=0.01, BF01=69.22; proactive familiarised versus wakeful rest - BF10=0.02, BF01=57.89). This suggests that any differences in arousal during the explore task prior to encoding, at least as captured by our ratings, were not the reason for the Proactive Resting effect we observed.

We also explored response times in the encoding task as a behavioural measure of arousal during that task itself, assuming slower responses if participants were more fatigued. As above, we selected response time data (measured in milliseconds) from the Day 2 encoding task, for only the proactive explore groups and wakeful rest group. We removed any responses less than 150ms on the basis that they were too quick for participants to have read the word stimulus on-screen, and then log-transformed the data to normalise them. However, we found that a linear model with an intercept only was favoured over one with experimental group as a predictor of the log-transformed response times (BF10=0.02, BF01=50.66), i.e. evidence that response times did not differ between the groups. This again supports the view that arousal, at least as now measured by reaction times, did not result in differences in attentiveness during encoding, providing no support for the arousal hypothesis as an explanation of the present Proactive Resting effect.

Of course, our self-reported arousal ratings probably lack reliability. In line with this view, 122 participants reported feeling highly aroused during the relaxation task, which seems implausible. The aforementioned group difference in arousal ratings, could be because participants made their ratings at the end of the session, so they were simply more likely to provide higher ratings for the explore task, having experienced the relatively less arousing relaxation task. If this were the case, then there may have been no true difference in arousal prior to encoding, which still does not explain the present results.

Overall, these data do not support the arousal hypothesis that pre-encoding activity modulates arousal which carries over to affect ability to encode words shortly afterwards.

### Lifestyle Qs

Only the final day of the experiment, participants were asked how often they play first-person-perspective or point-of-view (POV) video games, as we suspected that playing these types of games often could have reduced the novelty of the explore task. The question was given as follows: “Some video games are designed to be first-person perspective or 'point-of-view', where the user operating the controls feels like the character moving in the game. How often do you play these types of video games currently (within the past year)?” (4 response levels: “Never”, “Occasionally (e.g. 2-3 times per month)”, “2-3 times per week”, “Every day or almost every day”). 700 participants gave a response (of N=744). A Bayesian ordinal regression model confirmed that gaming frequency did not differ between groups (BF10=0.09, BF01=11.27). We divided our dataset into two subsamples: low-frequency gamers, who reported playing POV games a few times per month or less, of which there were only n=178, and high-frequency gamers, who reported playing POV games a few times per week or more, of which there were n=522. Recall data for these subsamples is shown in Figure S4.


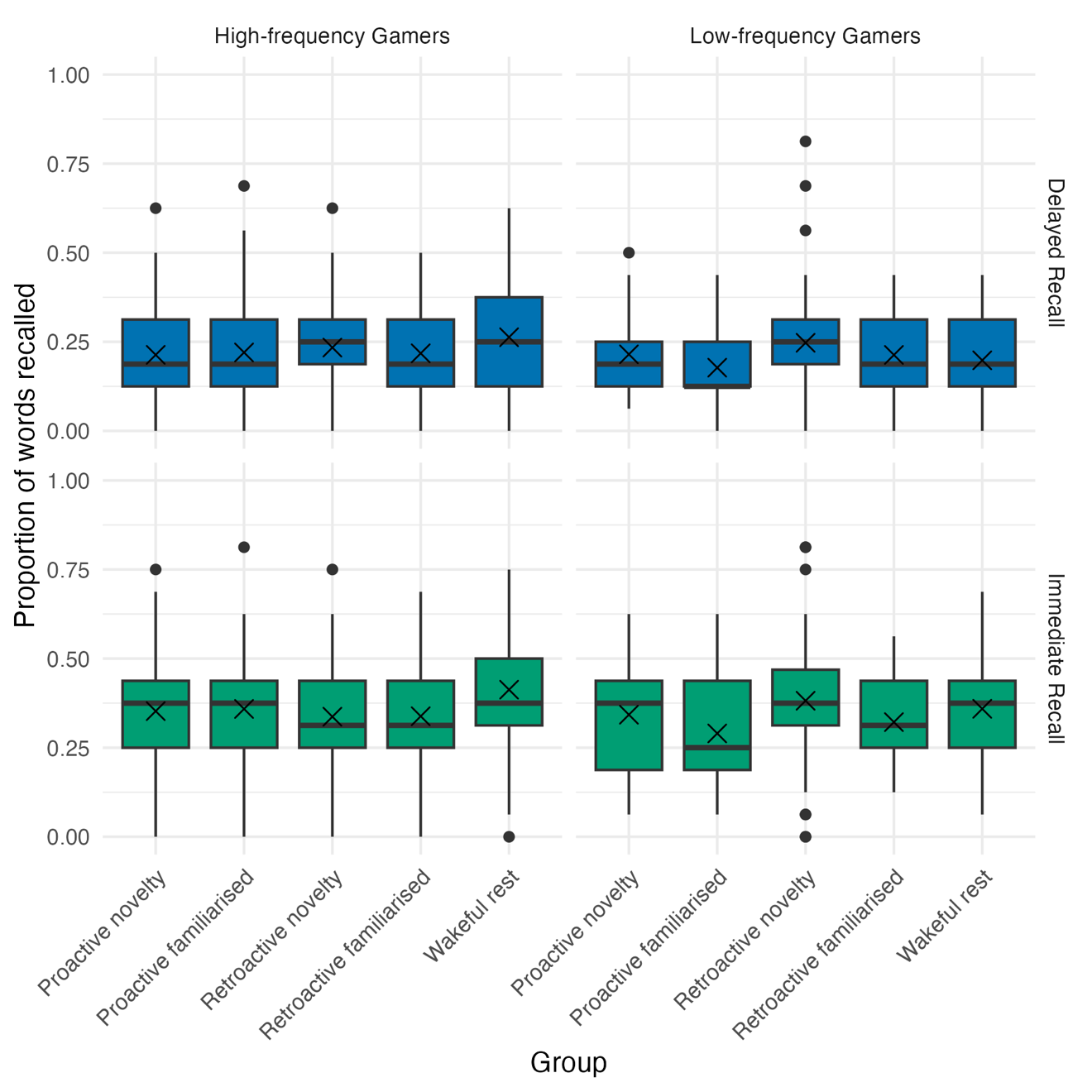


*Figure S4. Recall data split by gaming frequency. Group means represented with Xs. Note, the high-frequency gamers sub-sample is much larger (n=522) than the low-frequency gamers subsample (n=178).*

Whereas the high-frequency gamers’ pattern of recall data largely reflects that of the full sample (i.e. only evidence of a benefit of wakeful rest), the low-frequency gamers possibly show a trend towards a novelty effect (evident in the medians of the proactive groups and the medians and distributions of the retroactive groups). However, when comparing a model predicting recall from experimental group only, with a model containing an interaction between experimental group and gaming frequency (high/low), using data from the proactive and retroactive exploration groups only (since these were related to the novelty effect), we found strong evidence against an interaction between experimental group and gaming frequency for both immediate and delayed recall (BF10=0.06, BF01=17.28 and BF10<0.01, BF01=144.84, respectively). Thus, the apparent fluctuations in performance across conditions in the gamer vs non-gamer groups is likely to be due to noise, rather than the emergence of a novelty effect in non-gamers only. Overall, the large number of high-frequency gamers in this study does make it hard to discern whether playing POV video games often may interfere with a novelty effect, but the evidence seems to suggest that the lack of novelty effect in this study was not due to a moderating influence of playing POV games.
